# A neutrophil–epithelial thrombospondin‑1–CD36–c‑Jun N‑terminal kinase axis orchestrates mucosal barrier failure in intestinal ischemia–reperfusion

**DOI:** 10.1093/burnst/tkag059

**Published:** 2026-09-02

**Authors:** Yiwen Wang, Ran Li, Mengmeng Zhuang, Yuechen Fu, Qing Xu, Luqi Qiao, Simiao Chen, Jiayi Cao, Kehui Li, Ao Wang, Chao Meng, Wensheng Wang, Yong Sun

**Affiliations:** Department of Burn Surgery, The Affiliated Huaihai Hospital of Xuzhou Medical University, 226 Tongshan Street, Yunlong District, Xuzhou, Jiangsu Province 221004, China; Department of Burn Surgery, The 71st Group Army Hospital of People’s Liberation Army, 226 Tongshan Street, Yunlong District, Xuzhou, Jiangsu Province 221004, China; Department of Burn Surgery, The 71st Group Army Hospital of People’s Liberation Army, 226 Tongshan Street, Yunlong District, Xuzhou, Jiangsu Province 221004, China; Department of Burn Surgery, The Affiliated Huaihai Hospital of Xuzhou Medical University, 226 Tongshan Street, Yunlong District, Xuzhou, Jiangsu Province 221004, China; Department of Burn Surgery, The 71st Group Army Hospital of People’s Liberation Army, 226 Tongshan Street, Yunlong District, Xuzhou, Jiangsu Province 221004, China; Department of Burn Surgery, The Affiliated Huaihai Hospital of Xuzhou Medical University, 226 Tongshan Street, Yunlong District, Xuzhou, Jiangsu Province 221004, China; Department of Burn Surgery, The 71st Group Army Hospital of People’s Liberation Army, 226 Tongshan Street, Yunlong District, Xuzhou, Jiangsu Province 221004, China; Department of Burn Surgery, The Affiliated Huaihai Hospital of Xuzhou Medical University, 226 Tongshan Street, Yunlong District, Xuzhou, Jiangsu Province 221004, China; Department of Burn Surgery, The 71st Group Army Hospital of People’s Liberation Army, 226 Tongshan Street, Yunlong District, Xuzhou, Jiangsu Province 221004, China; Department of Burn Surgery, The Affiliated Huaihai Hospital of Xuzhou Medical University, 226 Tongshan Street, Yunlong District, Xuzhou, Jiangsu Province 221004, China; Department of Burn Surgery, The 71st Group Army Hospital of People’s Liberation Army, 226 Tongshan Street, Yunlong District, Xuzhou, Jiangsu Province 221004, China; Department of Burn Surgery, The Affiliated Huaihai Hospital of Xuzhou Medical University, 226 Tongshan Street, Yunlong District, Xuzhou, Jiangsu Province 221004, China; Department of Burn Surgery, The 71st Group Army Hospital of People’s Liberation Army, 226 Tongshan Street, Yunlong District, Xuzhou, Jiangsu Province 221004, China; Department of Burn Surgery, The Affiliated Huaihai Hospital of Xuzhou Medical University, 226 Tongshan Street, Yunlong District, Xuzhou, Jiangsu Province 221004, China; Department of Burn Surgery, The 71st Group Army Hospital of People’s Liberation Army, 226 Tongshan Street, Yunlong District, Xuzhou, Jiangsu Province 221004, China; Department of Burn Surgery, The Affiliated Huaihai Hospital of Xuzhou Medical University, 226 Tongshan Street, Yunlong District, Xuzhou, Jiangsu Province 221004, China; Department of Burn Surgery, The 71st Group Army Hospital of People’s Liberation Army, 226 Tongshan Street, Yunlong District, Xuzhou, Jiangsu Province 221004, China; Department of Burn Surgery, The Affiliated Huaihai Hospital of Xuzhou Medical University, 226 Tongshan Street, Yunlong District, Xuzhou, Jiangsu Province 221004, China; Department of Burn Surgery, The 71st Group Army Hospital of People’s Liberation Army, 226 Tongshan Street, Yunlong District, Xuzhou, Jiangsu Province 221004, China; Department of Burn Surgery, The 71st Group Army Hospital of People’s Liberation Army, 226 Tongshan Street, Yunlong District, Xuzhou, Jiangsu Province 221004, China; Department of Burn Surgery, The 71st Group Army Hospital of People’s Liberation Army, 226 Tongshan Street, Yunlong District, Xuzhou, Jiangsu Province 221004, China; Department of Burn Surgery, The Affiliated Huaihai Hospital of Xuzhou Medical University, 226 Tongshan Street, Yunlong District, Xuzhou, Jiangsu Province 221004, China; Department of Burn Surgery, The 71st Group Army Hospital of People’s Liberation Army, 226 Tongshan Street, Yunlong District, Xuzhou, Jiangsu Province 221004, China

**Keywords:** Intestinal ischemia–reperfusion injury, Neutrophils, Apoptosis, Thrombospondin-1, CD36, JNK signaling, Intestinal mucosal barrier

## Abstract

**Background:**

Intestinal barrier failure following ischemia–reperfusion (IR) injury is a critical event that drives lethal systemic complications; however, the specific intercellular signals governing epithelial cell fate under these pathological conditions remain incompletely understood.

**Methods:**

We performed single-cell ribonucleic acid sequencing (scRNA-seq) on intestinal tissues from a murine IR model to map intercellular communication networks. The identified ligand–receptor pair was functionally validated using neutrophil-specific thrombospondin-1 (*Thbs1*) knockout mice, combined with pharmacological inhibition and activation of CD36, as well as biochemical and histological analyses.

**Results:**

scRNA-seq analysis of the IR-injured gut revealed a key neutrophil–epithelial signaling pathway mediated by the *Thbs1*–*Cd36* ligand–receptor pair. Neutrophil-specific genetic ablation of *Thbs1* markedly attenuated IR-induced epithelial apoptosis and barrier disruption. Mechanistically, THBS1 binding to epithelial CD36 activated JNK signaling, mediating a proapoptotic cascade. CD36 inhibition was protective, whereas its activation bypassed the requirement for THBS1 and provoked mucosal injury.

**Conclusions:**

Our study elucidates a key mechanism of gut barrier failure and identifies the THBS1–CD36–JNK axis as a promising therapeutic target for mitigating intestinal IR injury in critical conditions such as burns and shock.

## Graphical Abstract

**Graphical Abstract:**
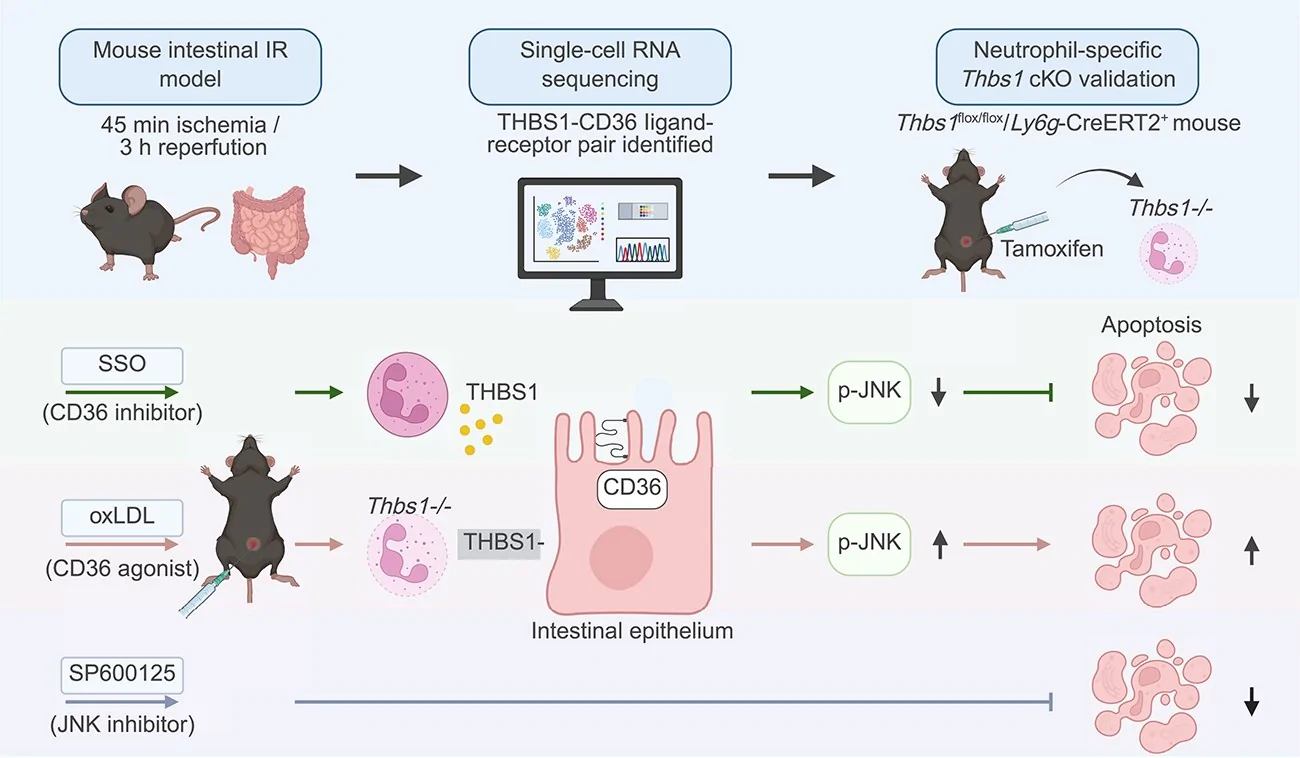

## Highlights

- Single-cell transcriptomics identifies ischemia-reperfusion-specific neutrophil–epithelial Thbs1–CD36 crosstalk.
- Neutrophil-specific Thbs1 deletion preserves intestinal barrier integrity and reduces epithelial apoptosis after ischemis-reperfusion injury.
- Epithelial CD36 activates JNK downstream of neutrophil-derived Thbs1 to drive epithelial cell death.
- CD36 activation bypasses Thbs1 deficiency, whereas CD36 or JNK inhibition mitigates mucosal injury.
- The Thbs1–CD36–JNK axis provides a targetable mechanism of intestinal barrier failure relevant to burns and shock.

## Background

The intestinal mucosal barrier serves as a critical interface between the host and the external environment, with its integrity being essential for the maintenance of systemic homeostasis [1–4]. This barrier is particularly vulnerable to ischemia–reperfusion (IR) injury—a common pathophysiological event in severe shock, major surgery and burns—in which its breach initiates bacterial translocation, systemic inflammation, and ultimately multiple organ dysfunction [5–12]. As the primary structural component of this barrier, intestinal epithelial cells (IECs) are highly susceptible to apoptosis under stress conditions [13–17]. While inflammatory mediators such as TNF-α and reactive oxygen species (ROS) are well-established inducers of IEC apoptosis [13, 18–21], the precise mechanisms through which infiltrating immune cells directly orchestrate epithelial cell death within the complex mucosal microenvironment remain inadequately explored.

Neutrophils are among the first immune responders to infiltrate the intestinal mucosa following injury, including in scenarios such as infectious peritonitis and severe burns [19–23]. During IR injury, they disrupt the epithelial barrier through multiple well-characterized mechanisms, including ROS-mediated oxidative damage, the secretion of proinflammatory cytokines (TNF-α and IL-1β), the release of proteases (elastase and matrix metalloproteinases), and the formation of neutrophil extracellular traps (NETs) [22–25]. However, these mechanisms largely involve indirect paracrine effects or nonspecific inflammatory amplification. Recent studies have revealed that neutrophils can also regulate intestinal inflammation through more direct cell–cell communication pathways, such as those involving PAD4–CKMT1 and elastase–E-cadherin signaling [26, 27]. Nevertheless, whether neutrophils engage in direct ligand–receptor interactions with IECs via specific secreted or membrane-bound molecules to precisely trigger epithelial cell death during early IR injury remains unknown.

Thrombospondin-1 (THBS1), a multifunctional glycoprotein with context-dependent roles in immunity and apoptosis, represents a candidate molecule of interest [28–30]. THBS1, an important extracellular matrix protein, contributes to repair processes in various tissues. Recent studies highlight its crucial role in intestinal mucosal repair. It regulates IEC migration and regeneration through receptors such as CD47 and transforming growth factor-β (TGF-β) [31]. In mouse models, the absence of THBS1 restricts epithelial migration and delays wound healing, thereby impairing barrier repair [32]. Complementary *in vitro* evidence confirms that THBS1 promotes epithelial migration and wound healing by modulating the activity of the RhoA and Rac1 signaling pathways [33]. However, it also exacerbates intestinal inflammation in colitis models by promoting macrophage differentiation and proinflammatory cytokine secretion [34]. This functional duality underscores the importance of identifying its key receptor partner in specific pathological contexts. Notably, the direct ligand–receptor relationship between THBS1 and CD36 has been established biochemically [35]. CD36 is expressed on both neutrophils and epithelial cells and is involved in diverse processes ranging from fatty acid uptake to inflammatory signaling [36, 37]. Importantly, the stress-activated kinase JNK—a well-established mediator of epithelial apoptosis in intestinal IR [38, 39]—has been shown to function downstream of CD36 in models of vascular biology and metabolic disease [40, 41]. However, whether a complete signaling axis connecting neutrophil-derived THBS1, epithelial CD36, and JNK exists in intestinal IR has not been established.

On the basis of these premises, we hypothesized that a THBS1–CD36–JNK signaling axis mediates neutrophil-driven IEC apoptosis during IR injury. To test this hypothesis, we used an unbiased discovery strategy involving single-cell ribonucleic acid sequencing (scRNA-seq) to map intercellular communication in the injured gut. This was followed by rigorous functional validation through *in vivo* neutrophil depletion and neutrophil-specific *Thbs1* knockout models combined with mechanistic investigations. Our results systematically revealed a previously unrecognized damage signaling network that includes neutrophils and epithelial cells, thereby addressing a critical mechanistic gap and revealing a targetable pathway to mitigate intestinal IR injury.

## Methods

### Experimental animals and genotyping

All animal experiments were conducted in compliance with institutional and national ethical guidelines and approved by the Animal Care and Use Committee of Xuzhou Medical University (Approval No. 202501 T012).

To generate neutrophil-specific *Thbs1* conditional knockout (*Thbs1*^flox/flox^/*Ly6g*-CreERT2^+^) mice, *Thbs1*-floxed mice (Cat# NM-CKO-2100206, Shanghai Model Organisms Center, China) were crossed with *Ly6g*-2A-CreERT2 mice (Cat# NM-KI-200218, Shanghai Model Organisms Center, China). Offspring were genotyped to identify animals carrying both the *Thbs1* floxed allele and CreERT2 transgene.

Genomic deoxyribonucleic acid (DNA) for genotyping was isolated from tail biopsies using a standard alkaline lysis method. Polymerase chain reaction (PCR) was performed on a ProFlex Base PCR System, with fragment sizes determined using the Vazyme DL 15000 DNA Marker (MD103). Representative genotyping images are presented in Figure S6a.

Male offspring carrying both the *Thbs1* floxed allele and the CreERT2 transgene were selected for experiments. Age-matched wild-type (WT) male C57BL/6 mice (6–8 weeks old, 20–22 g) from the Laboratory Animal Center of Xuzhou Medical University (production license: SCXK [Su] 2021-0006) served as controls.

Tamoxifen was dissolved in corn oil to prepare a 20 mg/ml stock solution, which was incubated overnight at 37°C with continuous shaking. To induce Cre-mediated recombination, *Thbs1* cKO mice received daily intraperitoneal injections of tamoxifen (75 mg/kg) for five consecutive days. Control animals (WT and/or vehicle-treated *Thbs1* cKO mice) received equivalent volumes of corn oil. Tamoxifen-treated and untreated mice were housed separately to prevent nonspecific knockout through social grooming. All subsequent experiments were conducted 7 days after the final injection to ensure complete gene deletion and tamoxifen clearance. The results of the validation of neutrophil purity are shown in Figure S6b.

All the animals were maintained under specific pathogen-free (SPF) conditions with free access to food and water and underwent one week of acclimation before the experimental procedures.

### Neutrophil depletion model

To achieve efficient neutrophil depletion, C57BL/6 mice received intraperitoneal injections of anti-Ly6G monoclonal antibody (clone 1A8; Bio X Cell; Cat# BE0075-1) at a dose of 500 μg per 20 g body weight, which was administered 24 h and again 4 h prior to the induction of intestinal IR injury. Following antibody administration, the mice were maintained under SPF conditions and monitored until the surgical procedures commenced. Neutrophil depletion efficiency was validated by flow cytometry. Peripheral blood was collected from the orbital sinus of the mice and incubated with erythrocyte sedimentation solution at room temperature for 40 min. The leukocyte-rich upper layer was carefully collected and centrifuged at 300 × g for 8 min. The supernatant was discarded, and the cell pellet was resuspended and adjusted to a concentration of ~1 × 10^7^ cells/ml. The cells were then stained with a PE-conjugated anti-mouse Ly6G antibody (Biolegend, Cat# 127607) and a FITC-conjugated anti-mouse/human CD11b antibody (Biolegend, Cat# 101206) at 4°C for 30 min in the dark. After staining, the cells were washed and resuspended in phosphate-buffered saline (PBS) for analysis. Samples were acquired on a BD FACSCanto™ II flow cytometer, and data were analyzed using FlowJo software (v10.0).

### Neutrophil isolation and coculture

For *in vitro* experiments, mouse peripheral blood neutrophils were collected via orbital sinus bleeding and isolated using a commercial neutrophil isolation kit (Beijing Solarbio Science & Technology Co., Ltd, Cat# P9201) following the manufacturer’s instructions. Briefly, freshly collected mouse anticoagulated blood was mixed with sterile PBS and erythrocyte sedimentation solution at a 1:1:1 ratio and incubated at room temperature for 40 min to allow erythrocyte sedimentation. The leukocyte-rich upper layer was carefully collected. Separation solution A and separation solution C were sequentially added to a centrifuge tube to form a density gradient, and the plasma layer was gently layered on top. Centrifugation was performed at 750 × g for 30 min at room temperature using a horizontal rotor with acceleration and deceleration set to 1. After centrifugation, the intermediate buffy coat layer (neutrophil layer) was collected, washed with cell washing solution, and centrifuged at 300 × g for 10 min. The supernatant was discarded, and the cell pellet was resuspended and adjusted to a concentration of ~1 × 10^7^ cells/ml.

Neutrophil purity was validated by flow cytometry using a PE-conjugated anti-mouse Ly6G antibody (Biolegend, Cat# 127607) and a FITC-conjugated anti-mouse/human CD11b antibody (Biolegend, Cat# 101206). After staining, the cells were washed, resuspended in PBS, and analyzed on a BD FACSCanto™ II flow cytometer. The data were processed using FlowJo software (v10.0). The results revealed that the neutrophil purity exceeded 90%. The validation results for neutrophil purity are presented in Supplementary Figure S7.

For coculture experiments, isolated neutrophils were added at 5 × 10^5^ cells per well to 6-well plates pre-seeded with MODE-K cells. Cocultures were maintained in RPMI 1640 medium supplemented with 10% serum and incubated at 37°C with 5% CO₂ for 24 h. After incubation, the cells were harvested for subsequent western blot analysis, cell viability assays, or apoptosis detection.

### Pharmacological interventions

*In vivo*: Pharmacological interventions were carried out at specified time points prior to intestinal IR surgery. The mice in the SSO group received a single intraperitoneal injection of the CD36 inhibitor sulfosuccinimidyl oleate (SSO; MedChemExpress; Cat# HY-108309) at 5 mg/kg 2 weeks before surgery. The inhibitor was prepared in saline containing 5% dimethyl sulfoxide (DMSO). The mice in the O group were administered a single intraperitoneal injection of the CD36 agonist oxidized low-density lipoprotein (oxLDL; Thermo Fisher Scientific; Cat# L34357) at 5 mg/kg 2 h before surgery, which was subsequently diluted in sterile PBS. Control mice received an equivalent volume of the corresponding vehicle following the same schedule and administration route.

*In vitro*: The mouse IEC line MODE-K was used for *in vitro* studies. To mimic the *in vivo* dosing regimen, SSO was added to the culture system 24 h before the initiation of coculture at a final concentration of 10 μM. oxLDL was added 2 h before coculture at a final concentration of 20 μg/ml. Control cells were treated with an equivalent volume of the corresponding vehicle at the same time points.

### Thrombospondin-1 protein stimulation

To evaluate the direct pathogenic effect of THBS1 on IECs, MODE-K cells were seeded in 6-well plates and cultured to ~70% confluence. Recombinant mouse THBS1 protein (R&D Systems, Cat# 7859-TH-050) was dissolved in sterile PBS and added to the culture medium at a final concentration of 1 μg/ml. The cells were incubated for 2 h at 37°C with 5% CO₂. After stimulation, the cells were harvested for subsequent western blot analysis, immunofluorescence (IF) staining, or cell viability assays. Control cells were treated with an equivalent volume of vehicle (sterile PBS) under the same conditions.

### c-Jun N-terminal kinase inhibitor treatment

For *in vivo* experiments, mice received a single intraperitoneal injection of the JNK inhibitor SP600125 (TargetMol, Cat# T2668) at a dose of 15 mg/kg body weight, dissolved in 5% DMSO in saline, 2 h prior to intestinal IR surgery. The control mice received an equivalent volume of vehicle (5% DMSO in saline) following the same schedule.

For *in vitro* experiments, MODE-K cells were preincubated with SP600125 at a final concentration of 10 μM for 2 h prior to coculture with neutrophils or stimulation with Thbs1 protein. The inhibitor was maintained throughout the coculture or stimulation period. Control cells were treated with an equivalent volume of vehicle (0.1% DMSO in culture medium).

### Single-cell and bulk ribonucleic acid sequencing data

scRNA-seq of murine intestinal tissues was performed using the BD Rhapsody™ platform (BD Biosciences, USA). The resulting in-house scRNA-seq dataset has been deposited in the Genome Sequence Archive (GSA) at the National Genomics Data Center, China National Center for Bioinformation, under accession number CRA041649 and is publicly available at https://ngdc.cncb.ac.cn/gsa/browse/CRA041649. To support and extend our findings, three publicly available murine scRNA-seq datasets (GSE138902, GSE135316, and GSE190581) were downloaded from the GEO database and used for data integration and comparative analysis. Additionally, two bulk RNA-seq datasets (GSE200320 and GSE73569) were retrieved from GEO to assist in differential expression and pathway enrichment analyses. The GSE37013 dataset was utilized for immune infiltration analysis.

### Single-cell ribonucleic acid sequencing data preprocessing and quality control

Following quality control, which included filtering cells with gene counts between 200 and 5000 and mitochondrial gene percentages below 10%, the UMI count matrix was log-normalized. The top 2000 variable genes were selected as integration anchors using the Seurat FindIntegrationAnchors function. Data integration was performed with the IntegrateData function. Principal component analysis (PCA) was conducted on the integrated data matrix to reduce dimensionality, with the first 50 principal components used for downstream analysis. Cell populations were identified using the FindClusters function at a resolution of 1, resulting in the categorization of cells into 39 distinct clusters visualized via uniform manifold approximation and projection (UMAP). The “FindAllMarkers” function was used to identify genetic markers, with thresholds set at a log fold change of 0.5, a minimum percentage difference of 0.25, and an adjusted *P* value <0.05.

### Cell–cell communication analysis

Cell–cell communication analysis was performed using the CellChat R package (v2.1.2) to investigate ligand–receptor interactions between IECs and immune populations within the scRNA-seq datasets, utilizing the CellChatDB.mouse database.

### Differential gene expression analysis

Differentially expressed genes (DEGs) were identified using the FindAllMarkers function in the Seurat package by applying a one-tailed Wilcoxon rank-sum test with Bonferroni-corrected adjusted *P* values. DEGs met the following criteria: a minimum log fold change of 0.5, a difference in the percentage of expressing cells of at least 0.2, and an adjusted *P* value <0.05.

### Protein–protein interaction network analysis

To explore potential interactions among the proteins encoded by the DEGs, all the DEGs were mapped to the STRING database. A confidence score threshold of ≥0.4 was applied, and the species was specified as *Mus musculus*. The network was constructed using Cytoscape software (v3.9.1), and node connectivity was analyzed via the cytoHubba plugin. Hub genes within the protein–protein interaction (PPI) network were selected on the basis of degree ranking.

### Cell culture and coculture

The mouse small IEC line MODE-K was purchased from Shanghai Yaji Biological Co., Ltd The cells were cultured in RPMI 1640 medium supplemented with 10% FBS (Clark Bioscience, China) at 37°C in an incubator with 5% CO_2_. To establish an intestinal monolayer barrier model, MODE-K cells were seeded at a density of 1 × 10^5^ cells per well into 6-well plates containing 3-μm polyester membrane Transwell inserts. The upper chamber contained 1 ml of culture medium, while the lower chamber contained 1.5 ml. The cells were cocultured for 24 h.

### Coimmunoprecipitation

To validate the interaction between THBS1 and CD36, coimmunoprecipitation (Co-IP) assays were performed. Cells from the coculture system were lysed in lysis buffer, and the lysates were centrifuged at 12 000 × g for 15 min at 4°C. The supernatants were subsequently collected. An aliquot of the total cell lysates was reserved as an input positive control. For immunoprecipitation, anti-CD36 antibody (Proteintech, Cat# 32731-1-AP) or normal rabbit IgG (Proteintech, Cat# 98136-1-RR) was preincubated with Protein A + G magnetic beads (Thermo Fisher Scientific, Cat# 88803) for 2 h at 4°C with rotation to generate antibody–bead conjugates. The antibody-conjugated beads were then incubated with the cell lysates overnight at 4°C with rotation. The next day, the beads were washed three times with lysis buffer and collected using a magnetic stand. Immunoprecipitated proteins were eluted by boiling in 2× sodium dodecyl sulfate-polyacrylamide gel electrophoresis (SDS–PAGE) loading buffer at 100°C for 10 min and stored at −20°C until further analysis. The immunoprecipitates and input samples were separated by SDS–PAGE and subjected to western blotting with anti-THBS1 antibody (Cell Signaling Technology, Cat# 37879), anti-CD36 antibody (Proteintech, Cat# 32731-1-AP), and anti-β-actin antibody (Proteintech, Cat# 20536-1-AP) to confirm the interaction and efficiency of immunoprecipitation.

### Histological analysis

Small intestinal tissues from C57BL/6 and *Thbs1*^flox/flox^/*Ly6g*-CreERT2^+^ mice were harvested, fixed in 4% paraformaldehyde, and embedded in paraffin. Sections were cut at a thickness of 4 μm. Following deparaffinization and rehydration, tissue sections were stained with hematoxylin and eosin (H&E). Histopathological damage to intestinal tissues was assessed using Chiu’s scoring system, which evaluates villus length, mucosal integrity, and inflammatory infiltration.

### Tissue immunofluorescence staining

Paraffin-embedded mouse small intestinal tissue sections were deparaffinized, rehydrated, and washed with PBS. After blocking with 1% bovine serum albumin (BSA), the sections were incubated overnight at 4°C with primary antibodies, including anti-occludin (Cell Signaling Technology, Cat# 91131 T; 1:300) and anti-tubulin (HUABIO, Cat# M1305-2; 1:1000) antibodies. After being washed, the sections were incubated with appropriate fluorophore-conjugated secondary antibodies, including goat anti-rabbit IgG (H + L) CoraLite^®^ Plus 488 (Proteintech, Cat# RGAR002; 1:1000) and goat anti-mouse IgG (H + L) CoraLite^®^ Plus 594 (Proteintech, Cat# RGAM004; 1:1000). The nuclei were counterstained with 4′,6-diamidino-2-phenylindole (DAPI). Images were acquired using an Olympus BX53 fluorescence microscope (Olympus, Tokyo, Japan).

### Cellular immunofluorescence staining

MODE-K cells were seeded into confocal-compatible culture dishes, fixed with 4% paraformaldehyde, and subjected to immunostaining without permeabilization to specifically detect proteins at the membrane and cell junctions. The same primary antibodies and dilutions as outlined above were used. Fluorescence images were captured using a Leica STELLARIS 5 laser scanning confocal microscope.

### Intestinal epithelial cell isolation

After the mice were euthanized, the small intestine was rapidly removed. A 5-cm segment was excised, opened longitudinally, and thoroughly rinsed with ice-cold sterile PBS to remove luminal contents. The intestinal segment was then cut into ~1-cm pieces and placed in prewarmed (37°C) digestion solution consisting of CMF-HBSS (KeyGEN, Cat# KGL2202-500) supplemented with 5 mM EDTA (Beyotime, Cat# C0196—100 ml), 25 mM HEPES (MedChemExpress, Cat# HY-D0857), and 10% fetal bovine serum (FBS). The tissue pieces were digested on a shaker at 37°C for 20 min. After digestion, the suspension was vigorously pipetted 20 times using a Pasteur pipette and filtered through a 200-mesh cell strainer. The filtrate was centrifuged at 300 × g for 6 min, and the resulting pellet was subsequently washed twice with PBS. The enriched IECs were then collected for subsequent protein extraction.

IEC purity was validated by flow cytometry using an APC-conjugated anti-mouse EpCAM antibody (Biolegend, Cat# 118214) and a PE-conjugated anti-mouse CD45 antibody (Biolegend, Cat# 147711). After staining, the cells were washed, resuspended in PBS, and analyzed on a BD FACSCanto™ II flow cytometer. The data were processed using FlowJo software (v10.0). The purity of isolated IECs from all the experimental groups is presented in Supplementary Figure S8.

### Western blot analysis

Mouse small intestinal tissues or MODE-K cells were homogenized or lysed in RIPA buffer containing protease inhibitors. The homogenates were centrifuged at 12 000 × g for 25 min at 4°C, and the supernatant was collected for total protein concentration determination using a BCA assay. Equal amounts of proteins were separated by SDS–PAGE, transferred to nitrocellulose membranes, and blocked with rapid blocking buffer. The membranes were incubated with specific primary antibodies overnight at 4°C, followed by incubation with the corresponding secondary antibodies at room temperature for 2 h. The immunoreactive bands were visualized using an Odyssey laser imaging system and analyzed with ImageJ software. For quantification of JNK phosphorylation, p-JNK band intensities were normalized to those of total JNK (t-JNK) from the same membrane, and the p-JNK/t-JNK ratio of each sample is expressed as the fold change relative to that of the control group. The membranes were incubated with the following primary antibodies: anti-GAPDH (Proteintech, Cat# 60004-1-Ig; 1:100000), anti-CD36 (Abmart, Cat# T55796; 1:2000), anti-beta-tubulin (Cell Signaling Technology, Cat# 2146S; 1:2000), anti-SAPK/JNK (Cell Signaling Technology, Cat# 9252 T; 1:2000), anti-P-SAPK/JNK (Cell Signaling Technology, Cat# 4668 T; 1:2000), anti-IL-6 (HUABIO, Cat# EM1701-45; 1:2000), anti-TNF-alpha (Proteintech, Cat# 80258-6-RR; 1:5000), and anti-occludin (Cell Signaling Technology, Cat# 91131 T; 1:2000) antibodies.

### Cell viability assay

Cell viability was assessed using the Cell Counting Kit-8 (CCK-8) assay (Beyotime, Shanghai, China) according to the manufacturer’s instructions. MODE-K cells were seeded in 96-well plates and pretreated with either the CD36 inhibitor SSO (10 μg/ml; MCE, Cat# HY-112847A) 24 h prior to coculture or the CD36 agonist oxLDL (50 μg/ml; Yiyuan Biotechnology, Guangzhou, China) 2 h before coculture. After treatment, MODE-K cells were cocultured with freshly isolated neutrophils under experimental conditions. At the end of the incubation period, 10 μL of CCK-8 solution was added to each well, and the plates were incubated at 37°C for 2 h. Absorbance was measured at 450 nm using a microplate reader (BioTek, Synergy H1, USA) to evaluate cell viability.

### Wound healing assay

A uniform wound was created in a confluent cell monolayer using a sterile 10 μL pipette tip. The cells were incubated in serum-free medium until wound closure. The wound area was observed and photographed at 0, 24, and 48 h using an inverted fluorescence microscope (Olympus IX73, Tokyo, Japan). The wound areas were measured using ImageJ software, and the cell migration rate was calculated as follows: [(Area at 0 h−Area at T h)/Area at 0 h] × 100%.

### Terminal deoxynucleotidyl transferase dUTP Nick End Labeling assay for assessment of apoptosis in tissues and cells

Apoptosis was evaluated using a terminal deoxynucleotidyl transferase dUTP nick end labeling (TUNEL) assay with a commercial kit (MA0223; Meilunbio, China) following the manufacturer’s instructions. For tissue sections, paraffin-embedded small intestinal sections were deparaffinized, rehydrated, and incubated with proteinase K (20 μg/ml) at 37°C for 30 min. After being washed with PBS, the sections were incubated in the dark with a TUNEL reaction mixture containing terminal deoxynucleotidyl transferase (TdT) and fluorescein-labeled dUTP for 60 min to label DNA strand breaks. The nuclei were counterstained with DAPI. Cells exhibiting red fluorescence were identified as TUNEL-positive apoptotic cells. Images were acquired using an Olympus BX53 upright fluorescence microscope (Olympus, Tokyo, Japan). For cultured MODE-K cells, the cells seeded in 6-well plates were fixed and incubated with proteinase K (20 μg/ml) at 37°C for 30 min. After being washed with PBS, the cells were treated with the TUNEL reaction mixture for 60 min in the dark. Following DAPI staining, apoptotic cells were visualized on the basis of red fluorescence using an inverted fluorescence microscope (Olympus IX73, Tokyo, Japan).

### Flow cytometry

Apoptosis was assessed using the Annexin V-FITC/PI Apoptosis Detection Kit (KeyGEN BioTECH, Jiangsu, China) according to the manufacturer’s instructions. Briefly, MODE-K cells were harvested using EDTA-free trypsin (Vicmed, Jiangsu, China), gently washed with PBS, and resuspended in binding buffer at a concentration of 1 × 10^5^ cells/100 μl. The cells were then stained with 5 μl of annexin V-FITC and 5 μl of propidium iodide (PI), incubated in the dark at room temperature for 15 min, and analyzed immediately using a BD FACSCanto™ II flow cytometer (BD Biosciences, USA). The percentage of apoptotic cells was calculated on the basis of the annexin V and PI staining patterns.

### Statistical analysis

Statistical analyses were performed using GraphPad Prism version 9.31. Data distribution was evaluated using the Shapiro–Wilk normality test, and homogeneity of variance was assessed using the Brown–Forsythe test. Normally distributed data with homogeneous variances are presented as the mean ± standard deviation, whereas nonnormally distributed data are presented as medians with interquartile ranges. For data meeting parametric assumptions, one-way, two-way, or three-way analysis of variance (ANOVA) was applied as appropriate, followed by Tukey’s post hoc test. For normally distributed data with unequal variances, Welch’s ANOVA followed by Dunnett’s T3 post hoc test was used where applicable. When the assumptions of normality and/or homogeneity of variance were not met, nonparametric tests were used: the Mann–Whitney U test for two independent groups and the Kruskal–Wallis test followed by Dunn’s multiple-comparison test for more than two groups. A *P* value <0.05 was considered to indicate statistical significance (*P < 0.05, **P < 0.01, and ***P < 0.001).

## Results

### Single-cell transcriptomic profiling reveals a neutrophil–epithelial Thrombospondin-1–CD36 signaling axis in intestinal IR injury

To map the cellular landscape of intestinal IR injury at single-cell resolution, we performed scRNA-seq on ileal tissues from a murine model of IR (45 min of ischemia/3 h of reperfusion) and sham-operated controls. Integrated analysis with UMAP dimensionality reduction resolved major intestinal cell populations, including IECs, goblet cells, T cells, macrophages, and neutrophils (Figure 1a). The results of the cell proportion analysis suggested an trend of expansion in the neutrophil population following IR treatment, although this difference did not reach statistical significance in our dataset (Figure 1b). To evaluate clinical relevance, we performed immune infiltration analysis on bulk RNA-seq data from human intestinal IR samples. Consistent with the trend observed in our murine model, the neutrophil enrichment scores were higher in human IR specimens (Figure 1c). We systematically analyzed cell–cell communication using the R package CellChat. Global analysis revealed that the overall interaction strength was greater in the IR group than in the sham group (Figure 1d). A chord diagram was used to visualize the overall communication patterns among different cell populations (Figure S1a). Defining neutrophils as ligand-sending cells for further analysis, we found that THBS signaling from these cells was significantly increased after IR (Figure S1b). A screening of differential ligand–receptor pairs revealed that the interaction between *Thbs1* (primarily expressed in neutrophils) and *Cd36* (mainly expressed in IECs) was among the most significantly upregulated communication pairs in the context of IR (Figure S1c). Similarly, *Cd36* mRNA expression levels in IECs significantly increased after IR treatment (Figure 1e).

**Figure 1:**
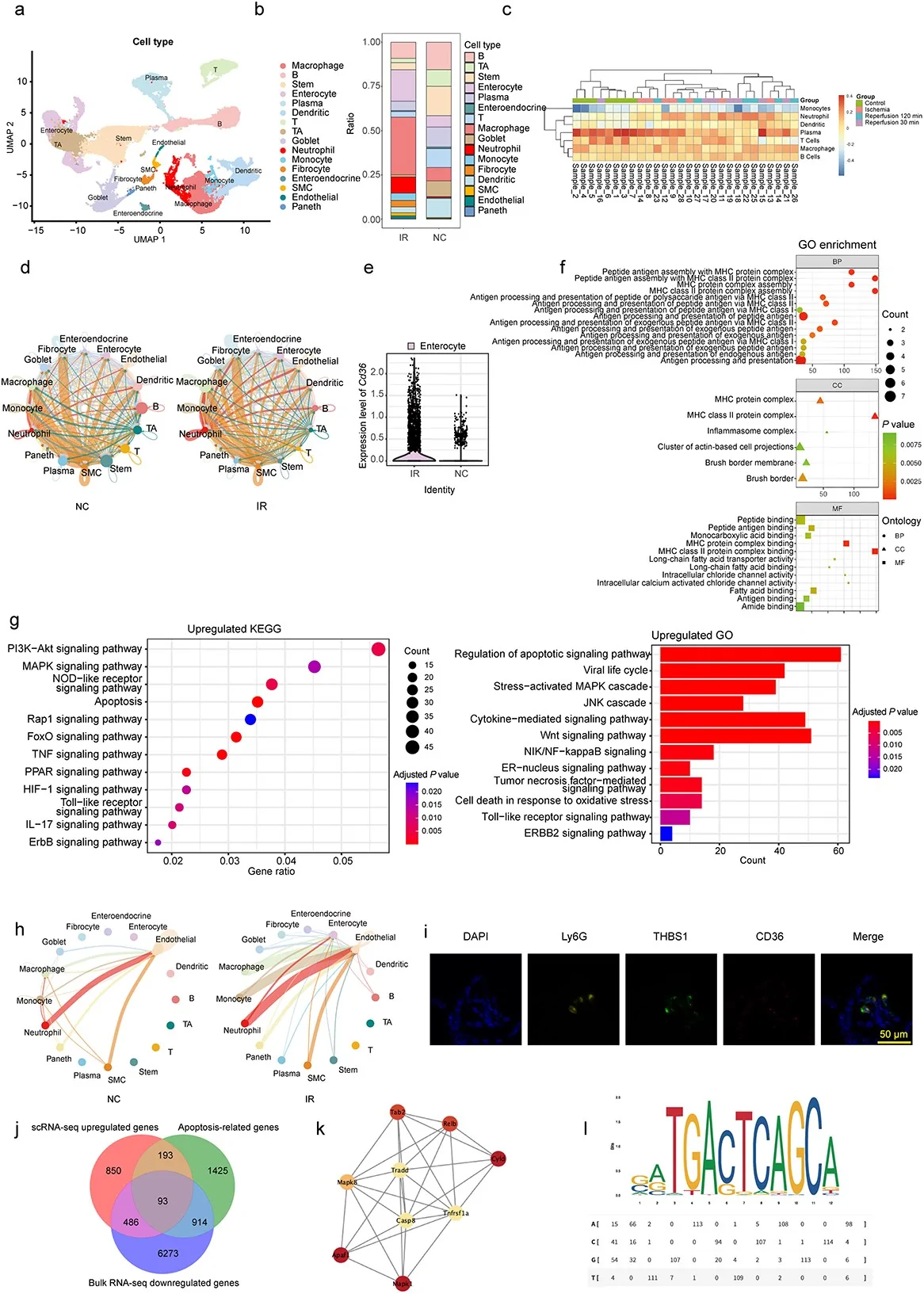
Single-cell transcriptomic profiling identifies a neutrophil–epithelial THBS1-CD36-JNK signaling axis in intestinal IR injury. (**a**) UMAP visualization of major intestinal cell populations in sham and IR groups. (**b**) Neutrophil proportion among all sequenced cells. (**c**) Neutrophil infiltration scores in human intestinal IR and control samples from bulk RNA-seq data. (**d**) Inferred cell–cell communication networks, showing enhanced overall interaction strength in IR. (**e**) CD36 expression levels in IECs by group. (**f**) GO biological process enrichment for downregulated genes in *Cd36*-knockout intestinal tissue. (**g**) Pathway enrichment analysis of upregulated genes in IR-injured IECs. (**h**) Specific enrichment of the THBS1-CD36 ligand-receptor pair between neutrophils and IECs in IR. (**i**) IF staining showing co-localization of THBS1 (green) with Ly6G^+^ neutrophils and CD36 (red) with adjacent IECs in IR tissues; nuclei were counterstained with DAPI (blue). Representative images from three independent experiments are shown (*n* = 3 mice per group). Scale bar: 50 μm. (**j**) Venn diagram of overlapping genes from integrated transcriptomic and apoptosis analyses. (**k**) PPI network of overlapping genes, with *Mapk8* (JNK1) identified as a top hub gene. (**l**) Transcription factor binding site prediction on the *Thbs1* promoter region. *IR* ischemia–reperfusion, *IECs* intestinal epithelial cells

To investigate the potential downstream functions of CD36, we analyzed published bulk RNA-seq data from the intestines of *Cd36* global knockout mice. Differential expression analysis revealed a distinct set of upregulated and downregulated genes in *Cd36* knockout intestines (Figure S1d). Gene Ontology (GO) enrichment analysis of the downregulated genes revealed significant enrichment in immune inflammation and apoptosis-related pathways (Figure 1f). Moreover, GO and Kyoto Encyclopedia of Genes and Genomes (KEGG) enrichment analysis of DEGs with upregulated expression in IR-injured mouse IECs indicated that these genes were significantly enriched in apoptosis and JNK signaling pathways (Figure 1g). We then returned to the cell communication analysis to specifically examine *Thbs1*–*Cd36* signaling, with neutrophils as ligand senders and IECs as receptor receivers. The results revealed that this specific signal was significantly enriched only in the IR group and was nearly absent in the sham group (Figure 1h). To validate the key neutrophil–epithelial ligand–receptor pair *Thbs1*–*Cd36* predicted by the bioinformatics analysis, we performed IF staining for spatial localization. In the IR model, we observed a clear THBS1 signal in Ly6G^+^ neutrophils, whereas the receptor CD36 was specifically expressed on adjacent IECs (Figure 1i). Notably, THBS1^+^ and CD36^+^ signals were colocalized along the intestinal epithelial barrier, suggesting that THBS1 derived from infiltrating neutrophils may bind to CD36 on epithelial cells and thereby act on the intestinal epithelium. To directly test this hypothesis, we performed Co-IP in a Transwell coculture system in which neutrophils were physically separated from MODE-K IECs. Following coculture, the cell lysates were immunoprecipitated with an anti-CD36 antibody. As shown in Figure S2, THBS1 was specifically coimmunoprecipitated with CD36 in the IR group, whereas no signal was detected in the IgG control group. These results provide direct biochemical evidence for the physical interaction between neutrophil-derived THBS1 and epithelial CD36, confirming the ligand–receptor relationship suggested by our spatial localization analysis.

On the basis of the pattern emerging from these complementary datasets—specifically, the IR-specific activation of the neutrophil–epithelial THBS1–CD36 axis coinciding with the proapoptotic state of epithelial cells and JNK pathway activation—we integrated three gene sets to identify potential mediator molecules: (i) upregulated genes in IR-injured epithelial cells from scRNA-seq; (ii) downregulated genes in CD36–KO intestines from bulk RNA-seq; and (iii) a known apoptosis-related gene set. Venn diagram analysis revealed 93 overlapping genes (Figure 1j). We constructed a PPI network from this gene set and applied a hub gene algorithm to rank nodes by importance, which revealed *Mapk8* (encoding JNK1) as one of the highest-connectivity nodes in the network (Figure 1k). Additionally, to preliminarily explore the upstream regulatory mechanisms controlling *Thbs1* expression in neutrophils, we performed transcription factor-binding site prediction on the *Thbs1* promoter region. The analysis revealed multiple potential binding sites, among which MAFK exhibited a high predictive binding score with well-conserved motifs (Figure 1l), suggesting its potential involvement in regulating *Thbs1* transcription in neutrophils under IR conditions.

Taken together, these complementary multiomics data suggest that neutrophil-derived THBS1 may drive apoptosis by activating the JNK signaling pathway via the epithelial CD36 receptor. These transcription factor prediction results also provide a potential avenue for future investigations into the upstream regulatory mechanisms of *Thbs1*.

### Neutrophil depletion alleviates IR-induced intestinal structural damage and inflammatory response

To define the pathogenic role of neutrophils in intestinal IR injury, we established three experimental groups: a control group (NC), an IR injury group, and a neutrophil-depleted group (NE) treated with anti-Ly6G antibody (1A8). NE mice received intraperitoneal injections of anti-Ly6G antibody 24 h and 4 h prior to IR surgery to specifically deplete circulating and tissue-infiltrating neutrophils (Figure 2a). All groups underwent 45 min of superior mesenteric artery ischemia followed by 3 h of reperfusion before sample collection. To rule out any potential effect of the neutrophil depletion procedure itself on intestinal homeostasis, we first validated the specificity of the anti-Ly6G antibody in sham-operated mice. The results revealed that treatment with anti-Ly6G antibody alone did not induce significant changes in intestinal length (Figure S3a) or intestinal tissue water content (Figure S3b) compared with those of the PBS-treated and isotype control groups. Furthermore, western blot analysis revealed that anti-Ly6G antibody treatment did not significantly affect the protein expression levels of the barrier protein occludin or the inflammatory cytokines IL-6 and TNF-α in intestinal tissues (Figure S3c–f). Flow cytometry analysis confirmed efficient neutrophil depletion following anti-Ly6G treatment (Figure S4). These results confirm that anti-Ly6G antibody-mediated neutrophil depletion does not, by itself, induce baseline intestinal alterations and that the protective effects observed subsequently are specifically attributable to the absence of neutrophils during injury. Having confirmed the safety of the depletion procedure, we next evaluated the effect of neutrophil depletion on IR injury. Macroscopic examination revealed that IR injury led to significant intestinal shortening (Figure 2b). We quantified this shortening by measuring the length from the stomach-duodenal junction to the cecum after a standardized folding procedure, which confirmed the significant reduction in the IR group (Figure 2c). Concurrently, IR injury exacerbated tissue edema, as indicated by a significant increase in the intestinal wet-to-dry weight ratio (Figure 2d). H&E staining revealed severe disruption of the intestinal mucosal architecture in the IR group, whereas neutrophil depletion markedly preserved tissue integrity (Figure 2e). Histological evaluation using Chiu’s scoring system further confirmed that IR induced substantial mucosal damage, and this pathology was significantly attenuated in neutrophil-depleted mice (Figure 2f). To assess intestinal epithelial barrier integrity, we examined the expression of the tight junction protein occludin. IF staining demonstrated that the occludin signal intensity was substantially reduced after IR injury but was partially restored in the neutrophil depletion group (Figure 2g). However, given that the intestinal mucosa comprises multiple cell types and that CD36 can be expressed on various immune cells, we further isolated primary IECs to determine whether the changes in protein expression occurred specifically in the epithelial cells themselves. Flow cytometric analysis revealed that the isolated cells exhibited a purity of >90% for the EpCAM^+^/CD45^−^ cells (Figure 2h), confirming successful enrichment of epithelial cells and effective exclusion of immune cell contamination. On this basis, we extracted total protein from the purified IECs for western blot analysis to precisely assess molecular alterations within the epithelial compartment. In these purified IEC lysates, IR injury resulted in significantly upregulated protein expression of the inflammatory cytokines IL-6 and TNF-α and downregulated expression of the barrier protein occludin in intestinal tissues. Concurrently, IR treatment elevated CD36 protein levels. In neutrophil-depleted mice, the aberrant overexpression of these inflammatory mediators and CD36 was suppressed, and the occludin protein levels were correspondingly restored (Figure 2i).

**Figure 2:**
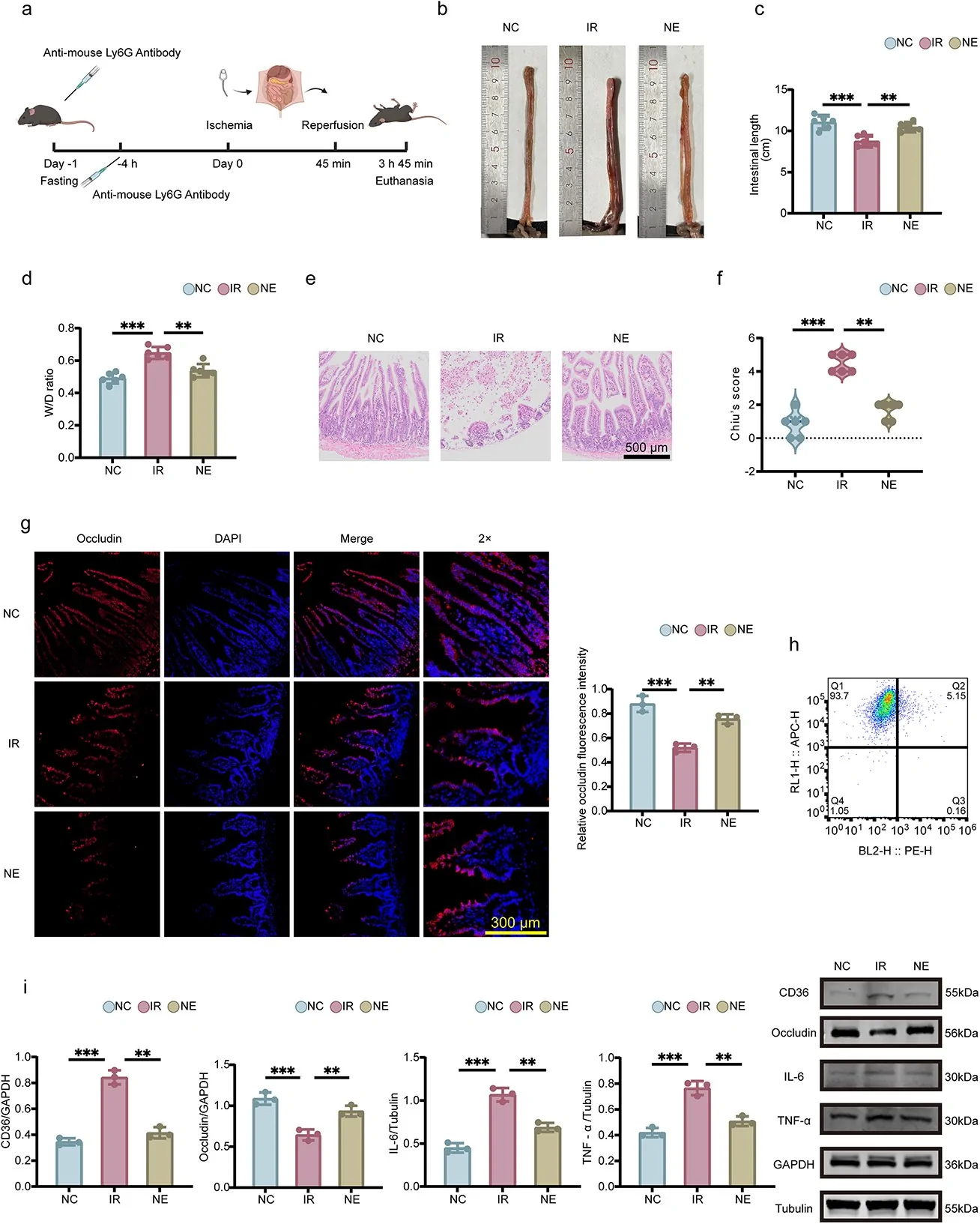
Neutrophil depletion alleviates IR-induced intestinal structural damage and inflammation. (**a**) Schematic of the experimental timeline for neutrophil depletion and intestinal IR injury. (**b**) Representative photographs showing the total intestinal length (*n =* 6 mice per group). (**c**) Statistical analysis of intestinal length across groups (*n =* 6 mice per group). (**d**) Intestinal wet/dry weight ratio indicating tissue edema (*n =* 6 mice per group). (**e**) Representative H&E-stained sections of intestinal tissues (*n =* 6 mice per group). (**f**) Histopathological assessment of intestinal injury by Chiu’s score (*n =* 6 mice per group). Scale bar: 500 μm. (**g**) IF staining (left) and quantification (right) of occludin (red) in intestinal epithelium, nuclei are stained with DAPI (blue) (*n =* 3 mice per group). Scale bar: 300 μm. (**h**) Flow cytometry analysis of IEC purity following isolation. A representative plot from one experimental group is shown, with complete data presented in Supplementary Figure S8 (*n =* 3 mice per group). (**i**) Western blot analysis of CD36, occludin, IL-6, and TNF-α expression in intestinal tissues (*n =* 3 mice per group). Groups: NC, sham-operated control; IR, ischemia–reperfusion; NE, IR + anti-Ly6G (neutrophil-depleted group). ^**^P < 0.01, and ^***^P < 0.001

Collectively, these *in vivo* findings demonstrate that neutrophil depletion significantly mitigates IR-induced intestinal structural damage, barrier dysfunction, and inflammatory activation.

### Neutrophil direct coculture induces epithelial barrier dysfunction and inflammatory activation

To directly validate the cytotoxic effects of neutrophils on IECs *in vitro*, we established a coculture system in which neutrophils isolated from mouse peripheral blood were cocultured with mouse IECs (MODE-K) for 24 h (Figure 3a). The experimental setup included a negative control group (NC; cocultured with neutrophils from sham-operated mice) and an IR group (cocultured with neutrophils from IR-injured mice). Calcein-AM/PI live-dead double staining revealed that coculture with the IR-group neutrophils significantly increased the proportion of PI-positive dead MODE-K cells (Figure 3b). Scratch wound assays demonstrated that IR-derived neutrophils markedly inhibited the migration ability of MODE-K cells (Figure 3c), which was further confirmed by quantitative analysis of migration rates (Figure 3d). CCK-8 assays indicated that the viability of MODE-K cells was significantly suppressed after coculture with IR-group neutrophils (Figure 3e). We subsequently evaluated changes in epithelial barrier integrity. IF staining revealed a reduction in the fluorescence signal of the tight junction protein occludin in MODE-K cells following coculture with IR-group neutrophils (Figure 3f). Quantitative analysis of fluorescence intensity confirmed the significant downregulation of the expression of occludin (Figure 3g). Western blot analysis further validated these phenotypic changes at the molecular level. Coculture with IR-group neutrophils led to significant upregulation of the expression of the proinflammatory cytokines IL-6 and TNF-α and downregulation of the expression of the barrier protein occludin in MODE-K cells. Concurrently, CD36 protein expression was significantly elevated (Figure 3h).

**Figure 3:**
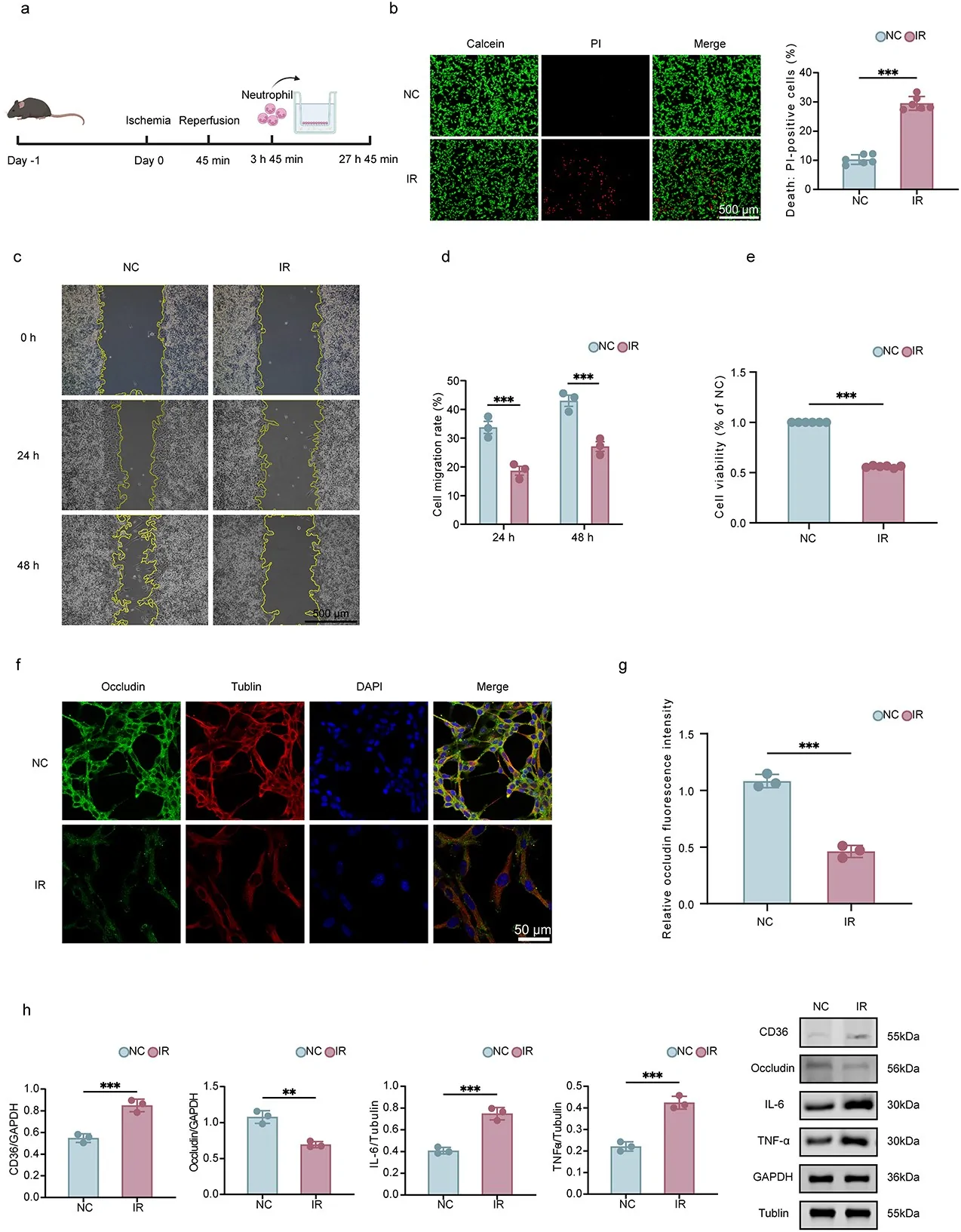
Neutrophil coculture induces epithelial barrier disruption and inflammatory activation. (**a**) Schematic of the coculture experiment. (**b**) Cell viability and death double staining (Calcein-AM/PI, left) and quantification of PI-positive cells (right) (*n =* 6 biological replicates). Scale bar: 500 μm. (**c**) Wound healing assay of MODE-K cells; representative images from three independent experiments are shown (*n =* 3 biological replicates). Scale bar: 500 μm. (**d**) Quantification of wound healing assay (*n =* 3 biological replicates). (**e**) MODE-K cell viability measured by CCK-8 assay (*n =* 6 biological replicates). (**f**) IF staining of occludin (green) in MODE-K cells, nuclei are stained with DAPI (blue) (*n =* 3 biological replicates). Scale bar: 50 μm. (**g**) Quantification of occludin IF intensity (*n =* 3 biological replicates). (**h**) Western blot analysis (upper) and densitometric quantification (lower) of CD36, occludin, IL-6, and TNF-α expression in MODE-K cells (*n =* 3 biological replicates). Groups: NC, cocultured with neutrophils from sham-operated mice; IR, cocultured with neutrophils from IR (ischemia–reperfusion)-injured mice. ^**^P < 0.01, and ^***^P < 0.001

These results demonstrate that neutrophils in the IR injury microenvironment can directly induce IEC death, suppress cell migration and viability, disrupt barrier integrity, and activate inflammatory responses. The concomitant upregulation of CD36 protein expression suggests its potential role in neutrophil-mediated epithelial injury.

### Neutrophil-specific knockout of Thrombospondin-1 alleviates intestinal mucosal damage induced by IR injury

To investigate the specific role of neutrophil-derived THBS1 in intestinal IR injury, we utilized *Thbs1*^flox/flox^/*Ly6g*-CreERT2^+^ mice to establish a neutrophil-specific *Thbs1* knockout model via tamoxifen induction. The mice then underwent intestinal IR treatment (45 min of ischemia/3 h of reperfusion). The experimental groups included a control group (NC), an IR injury group, a *Thbs1* knockout control group (T), and a *Thbs1* knockout + IR group (T + I). Intestinal tissues were collected after reperfusion for subsequent analysis (Figure 4a). Macroscopic analysis revealed significant shortening of intestinal length in the IR-treated mice (Figure 4b), which was further confirmed by quantitative assessment (Figure 4c). Concurrently, the intestinal wet weight/dry weight (W/D) ratio significantly increased in the IR group, indicating aggravated tissue edema; this parameter was partially restored in the T + I group (Figure 4d). Histological evaluation revealed severe disruption of the intestinal mucosal structure in the IR group, with H&E staining revealing villus shedding and disorganized glandular architecture (Figure 4e). Semiquantitative analysis using the Chiu scoring system confirmed that mucosal injury was significantly attenuated in the T + I group compared with the IR group (Figure 4f). To assess epithelial barrier integrity, we examined the expression of the tight junction protein occludin. IF staining revealed a marked reduction in the occludin (red) signal following IR treatment, while its expression was partially recovered in the T + I group (Figure 4g). Quantitative analysis of fluorescence intensity further supported these observations (Figure 4h). Western blot analysis of purified IEC lysates further validated these findings at the molecular level: compared with the IR group, the T + I group exhibited significantly decreased protein levels of the inflammatory cytokines IL-6 and TNF-α and restored expression of occludin. Moreover, the IR-induced upregulation of CD36 protein expression was notably suppressed in the T + I group (Figure 4i and j).

**Figure 4:**
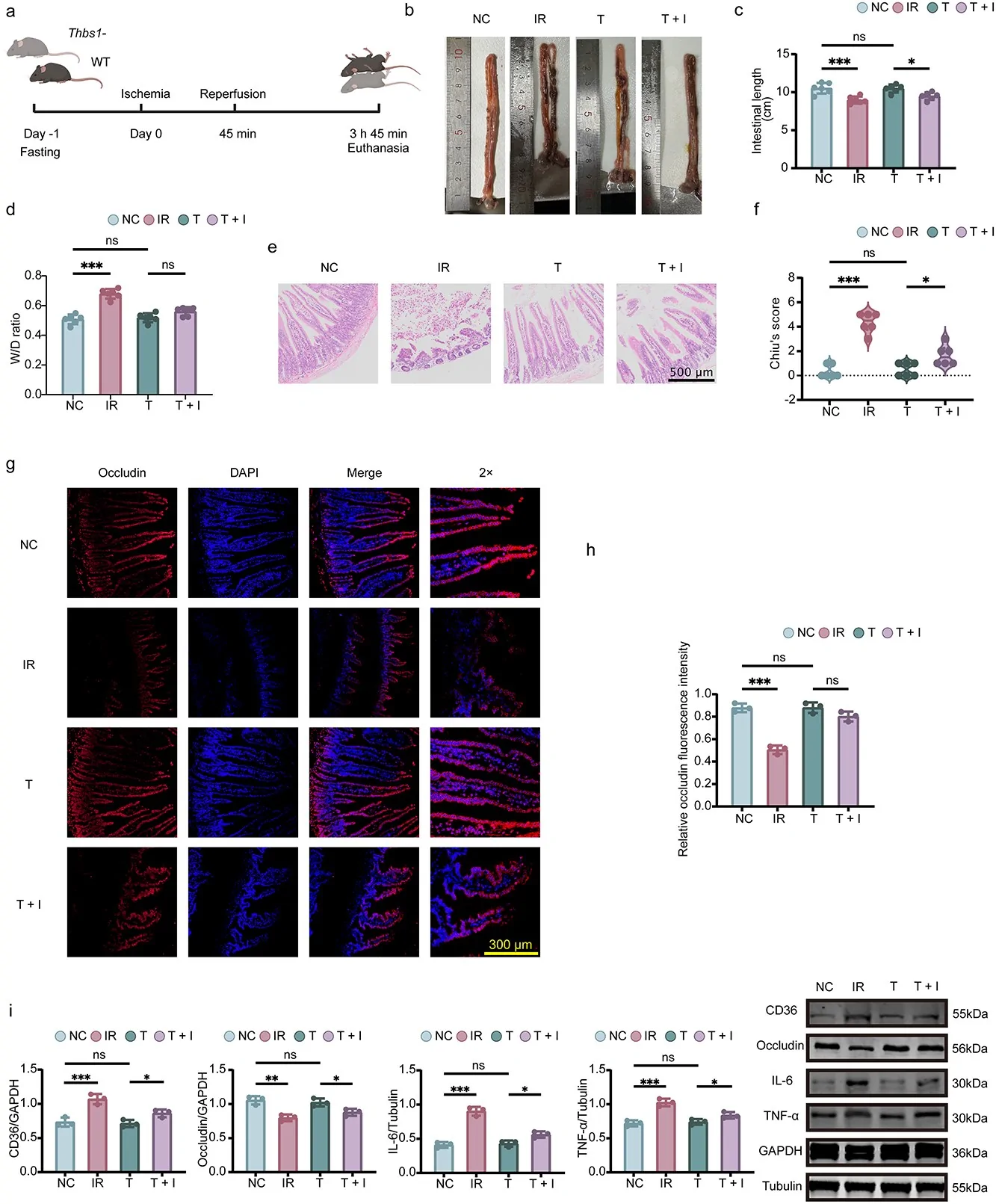
Neutrophil-specific knockout of Thbs1 alleviates IR-induced intestinal mucosal injury. (**a**) Experimental design and treatment timeline. (**b**) Representative photographs of intestinal length (*n =* 6 mice per group). (**c**) Statistical analysis of intestinal length across groups (*n =* 6 mice per group). (**d**) Quantification of intestinal wet/dry weight ratio (*n =* 6 mice per group). (**e**) Representative H&E-stained sections of intestinal tissues (*n =* 6 mice per group). Scale bar: 500 μm. (**f**) Histopathological evaluation of intestinal injury by Chiu’s score (*n =* 6 mice per group). (**g**) IF staining of occludin (red) in intestinal epithelium. Nuclei are stained with DAPI (blue) (*n =* 3 mice per group). Scale bar: 300 μm. (**h**) Quantification of occludin IF intensity (*n =* 3 mice per group). (**i**) Western blot analysis of CD36, occludin, IL-6, and TNF-α expression in intestinal tissues (*n =* 3 mice per group). (**j**) Densitometric quantification of western blot results (*n =* 3 mice per group). Groups: NC, sham-operated control; IR, ischemia–reperfusion; T, *Thbs1*^flox/flox^/*Ly6g*-CreERT2^+^ control; T + I, *Thbs1*^flox/flox^/*Ly6g*-CreERT2^+^ + IR. ^*^P < 0.05, ^**^P < 0.01, and ^***^P < 0.001

These results indicate that neutrophil-specific knockout of *Thbs1* effectively mitigates IR-induced intestinal structural damage, edema, barrier disruption, and inflammatory responses and suggest that CD36 may act as a downstream signaling molecule of THBS1 in this process.

### Neutrophil-derived Thrombospondin-1 directly mediates intestinal epithelial barrier dysfunction and inflammatory activation

To directly validate the role of neutrophil-derived THBS1 *in vitro*, we isolated neutrophils from the peripheral blood of four experimental mouse groups—control (NC), IR, *Thbs1* knockout control (T), and *Thbs1* knockout + IR (T + I)—and cocultured them with MODE-K cells (Figure 5a). Live-dead staining revealed that neutrophils derived from the IR group significantly increased MODE-K-cell death, an effect that was markedly reversed when neutrophils from *Thbs1* knockout mice (T + I) were used (Figure 5b). Statistical analysis further confirmed the significant differences in the proportion of PI-positive cells (Figure 5c). CCK-8 assays revealed that the absence of neutrophil-derived THBS1 significantly alleviated the inhibitory effect on MODE-K cell viability (Figure 5d). Scratch wound assays revealed that neutrophils in the IR group strongly suppressed cell migration, whereas the migratory capacity was substantially restored in the T + I group (Figure 5e). IF staining and quantitative analysis revealed a marked reduction in occludin expression in MODE-K cells cocultured with neutrophils from the IR group, whereas occludin expression was effectively restored in the T + I group (Figure 5f). Western blot analysis further corroborated these findings: compared with the IR group, the T + I coculture group exhibited significantly downregulated expression of the inflammatory cytokines IL-6 and TNF-α and recovery of the barrier protein occludin and notably suppressed the upregulation of CD36 protein expression (Figure 5g and h). These results demonstrate that neutrophil-derived THBS1 directly mediates cytotoxic effects on IECs and that its absence significantly alleviates epithelial barrier dysfunction and inflammatory activation. The observed changes in CD36 expression further suggest its potential role as a downstream molecule in THBS1-mediated processes. To evaluate the intrinsic pathogenic potential of the THBS1 protein alone, we directly stimulated MODE-K cells with purified THBS1 protein (TP group) and compared them with the NC, IR, T, and T + I groups. CCK-8 assays revealed that THBS1 protein stimulation significantly inhibited cell viability, with an effect comparable to that of coculture with neutrophils in the IR group, whereas cell viability was restored in the T + I group (Figure S5a). Western blot analysis further revealed that THBS1 protein stimulation significantly upregulated the expression of IL-6 and TNF-α and downregulated the expression of occludin, resulting in a phenotype similar to that of the IR group (Figure S5b–e). Notably, all these parameters were significantly restored in the T + I group. Statistical analysis revealed that the differences between the TP group and the T + I group were significant, confirming that THBS1 protein alone is sufficient to recapitulate the epithelial injury induced by IR-derived neutrophils. These results further support that THBS1 itself possesses intrinsic pathogenic potential.

**Figure 5:**
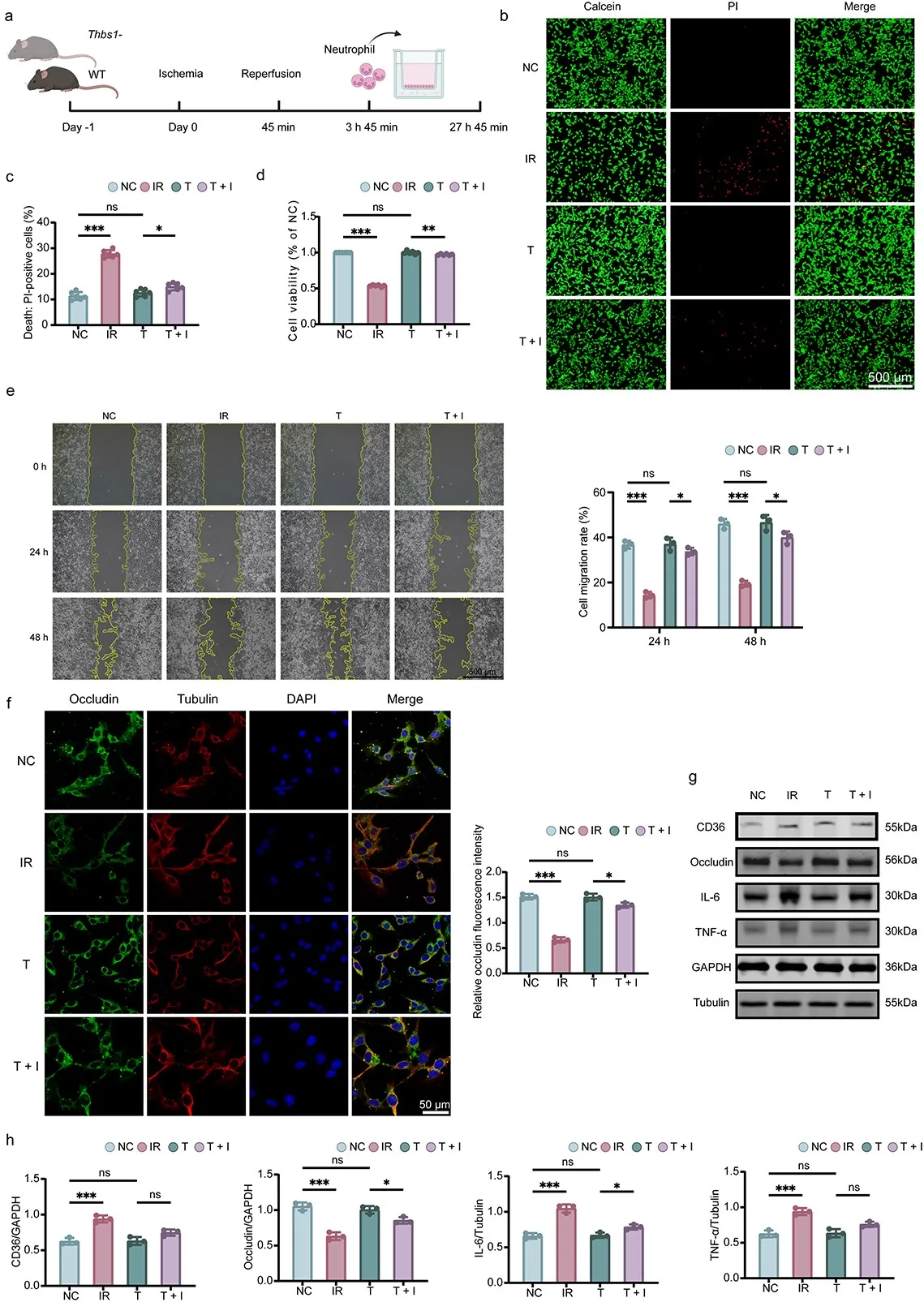
Neutrophil-derived THBS1 mediates intestinal epithelial barrier disruption and inflammatory activation. (**a**) Schematic of the coculture experiment using neutrophils from WT or *Thbs1*^flox/flox^/*Ly6g*-CreERT2^+^ mice. (**b**) Cell viability and death double staining (Calcein-AM/PI) (*n =* 6 biological replicates). Scale bar: 500 μm. (**c**) Quantification of PI-positive cells (*n =* 6 biological replicates). (**d**) MODE-K cell viability measured by CCK-8 assay (*n =* 6 biological replicates). (**e**) Wound healing assay (left) and quantification of migration rate (right) (*n =* 3 biological replicates). Scale bar: 500 μm. (**f**) IF staining of occludin (left) and quantification of fluorescence intensity (right) (*n =* 3 biological replicates). Scale bar: 50 μm. (**g**) Western blot analysis of CD36, occludin, IL-6, and TNF-α expression (*n =* 3 biological replicates). (**h**) Densitometric quantification of western blot results (*n =* 3 biological replicates). Groups: NC, cocultured with neutrophils from sham-operated mice; IR, cocultured with neutrophils from IR (ischemia–reperfusion)-injured mice; T, cocultured with neutrophils from *Thbs1*^flox/flox^/*Ly6g*-CreERT2^+^ control mice; T + I, cocultured with neutrophils from *Thbs1*^flox/flox^/*Ly6g*-CreERT2+ + IR mice. ^*^P < 0.05, and ^***^P < 0.001

### Thrombospondin-1 exacerbates ischemia–reperfusion-induced intestinal mucosal injury via CD36–JNK pathway-mediated intestinal epithelial cell apoptosis

To investigate whether THBS1 mediates IEC apoptosis through the CD36–JNK pathway, we established six experimental groups: control (NC), IR, CD36 inhibitor SSO treatment (S), *Thbs1*^flox/flox^/*Ly6g*-CreERT2^+^ control (T), *Thbs1* knockout + IR (T + I) and *Thbs1* knockout + IR + CD36 agonist oxLDL (O) (Figure 6a). All CreERT2-positive mice underwent tamoxifen-induced myeloid cell-specific *Thbs1* knockout. Intestinal tissues were collected immediately after the IR treatment, which consisted of 45 min of ischemia followed by 3 h of reperfusion. Macroscopic analysis revealed significant intestinal shortening in the IR group, which was effectively alleviated in the S group. This protective effect was reversed in the O group (Figure 6b). Analysis of the intestinal wet-to-dry weight ratio indicated that S treatment ameliorated IR-induced tissue edema, whereas O treatment partially counteracted the improvement conferred by *Thbs1* knockout (Figure 6c). Histological evaluation revealed severe mucosal damage and significantly elevated Chiu scores in the IR group. Both the T + I and S groups exhibited marked tissue protection, whereas injury severity was greater in the O group than in the T + I group (Figure 6d). IF staining and quantitative analysis demonstrated a significant IR-induced reduction in intestinal occludin expression, which was restored in the T and S groups. However, in the O group, occludin expression was further reduced (Figure 6e and f). Western blot analysis of purified IEC lysates revealed that IR injury significantly upregulated the protein expression levels of p-JNK, CD36, and the inflammatory cytokines TNF-α and IL-6 and downregulated expression of occludin. S treatment effectively suppressed these aberrant expression changes. In the T + I group, oxLDL treatment (O group) restored CD36 expression and reactivated JNK signaling (Figure 6g and h).

**Figure 6:**
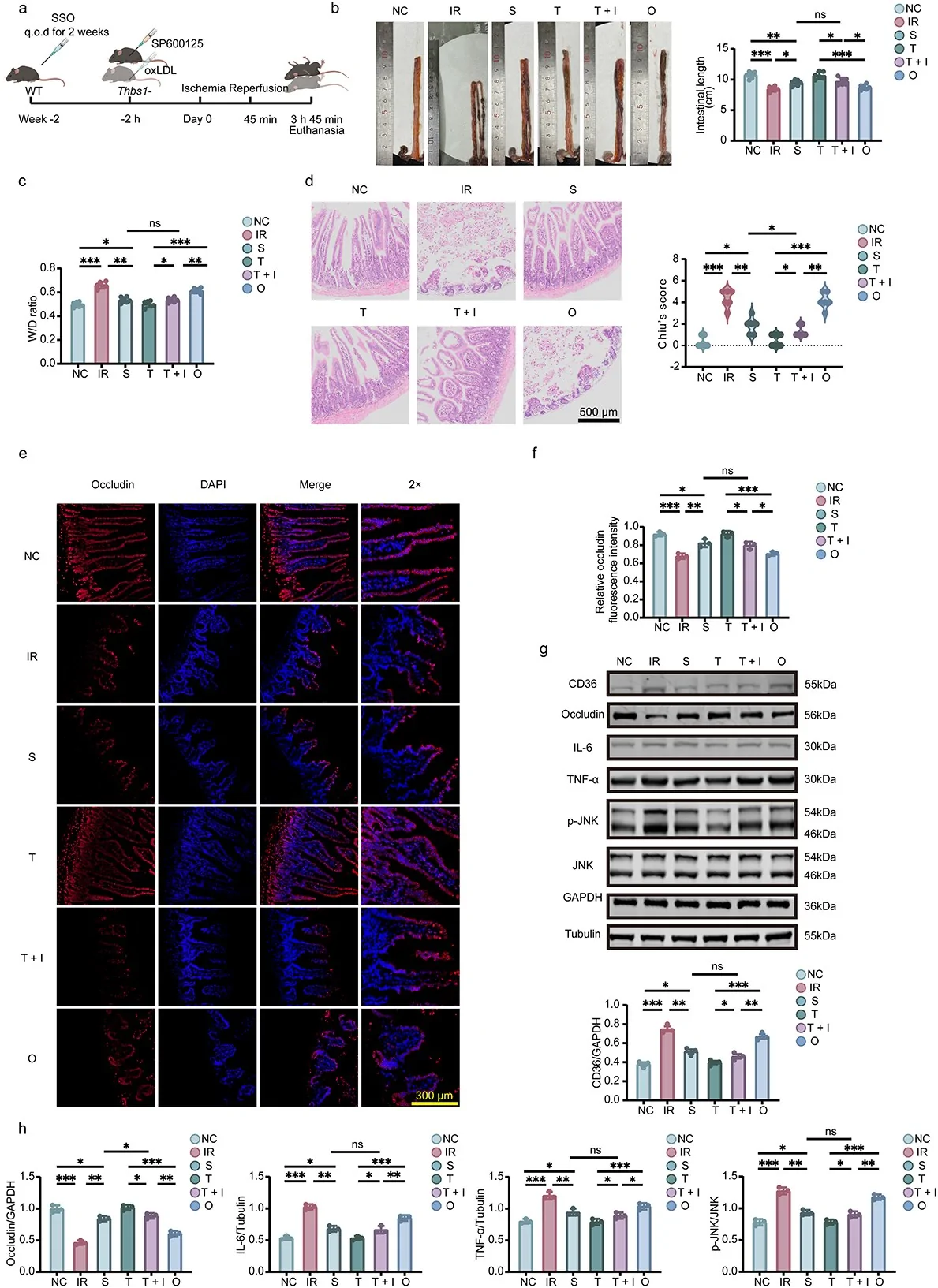
Activation of the CD36-JNK pathway by THBS1 promotes intestinal injury. (**a**) Experimental timeline of pharmacological interventions in WT and *Thbs1*^flox/flox^/*Ly6g*-CreERT2^+^ mice. (**b**) Measurement (left) and statistical analysis (right) of intestinal length across groups (*n =* 6 mice per group). (**c**) Quantification of intestinal wet/dry weight ratio (*n =* 6 mice per group). (**d**) Representative H&E-stained images (left) and histopathological evaluation by Chiu’s score (right) (*n =* 6 mice per group). Scale bar: 500 μm. (**e**) IF staining of occludin (red) in intestinal epithelium. Nuclei are stained with DAPI (blue) (*n =* 3 mice per group). Scale bar: 300 μm. (**f**) Quantification of occludin IF intensity (*n =* 3 mice per group). (**g**) Western blot analysis of CD36, occludin, IL-6, TNF-αand p-JNK, and expression and western blot analysis of CD36 (*n =* 3 mice per group). (**h**) Western blot analysis of occludin, IL-6, TNF-α, and p-JNK expression (*n =* 3 mice per group). Groups: NC, sham-operated control; IR, ischemia–reperfusion; S, IR + SSO (CD36 inhibitor); T, *Thbs1*^flox/flox^/*Ly6g*-CreERT2^+^ control; T + I, *Thbs1*^flox/flox^/*Ly6g*-CreERT2^+^ + IR; O, *Thbs1*^flox/flox^/*Ly6g*-CreERT2^+^ + IR + oxLDL (CD36 agonist). ^*^P < 0.05, ^**^P < 0.01, and ^***^P < 0.001

To determine whether IR-induced JNK activation and subsequent apoptosis occur in IECs, we performed IF colocalization analysis. Triple staining of p-JNK, TUNEL, and the epithelial marker E-cadherin revealed that both p-JNK and TUNEL signals were predominantly localized within E-cadherin-positive IECs (Figure 7a). To further elucidate the temporal relationship between JNK activation and apoptosis, we examined p-JNK and TUNEL signals at various time points following IR injury. As shown in Figure 7b, p-JNK-positive signals were detectable as early as 5 min after reperfusion, whereas TUNEL-positive cells began to appear at 30 min after reperfusion, confirming that JNK activation precedes epithelial apoptosis. Next, we evaluated the functional role of JNK in THBS1–CD36-induced apoptosis. TUNEL staining (Figure 7c) and quantitative analysis (Figure 7d) revealed that IR injury significantly increased the number of apoptotic epithelial cells, whereas this effect was markedly reduced by CD36 inhibitor (SSO) treatment or *Thbs1* knockout (T + I). Notably, oxLDL treatment reversed the antiapoptotic effect conferred by *Thbs1* knockout. To directly assess the necessity of JNK in this process, we included a JNK inhibitor group (SP, IR + SP600125). As shown in Figure 7e, JNK inhibitor treatment significantly reduced IR-induced apoptosis, with a protective effect comparable to that of CD36 inhibition.

**Figure 7:**
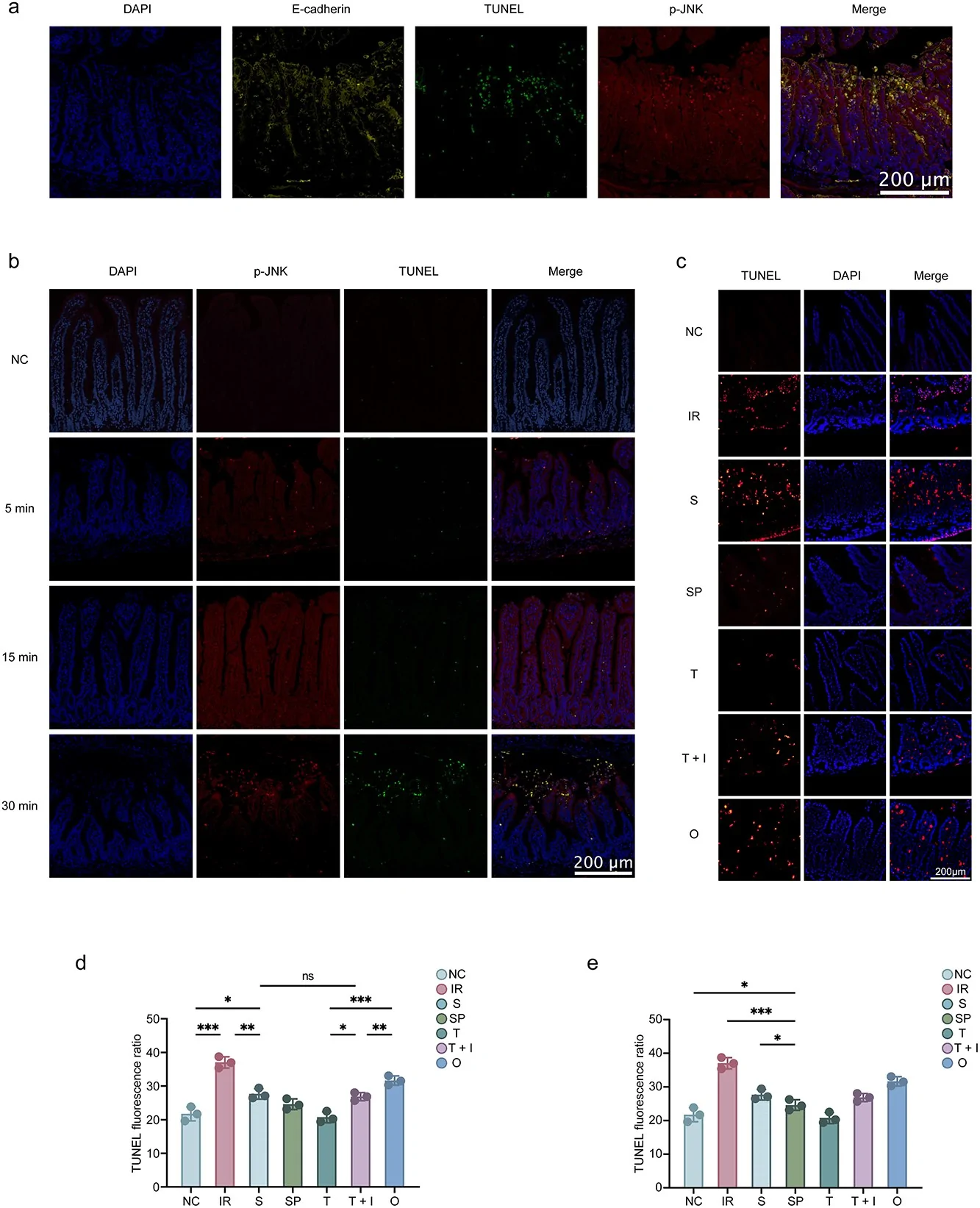
TUNEL staining confirms THBS1/CD36/JNK-mediated epithelial apoptosis *in vivo*. (**a**) IF co-localization of p-JNK (red), TUNEL (green), and E-cadherin (yellow), nuclei were stained with DAPI (blue) (*n =* 3 mice per group). Scale bar: 200 μm. (**b**) Temporal analysis of p-JNK (red) and TUNEL (green) at 5 min and 30 min after reperfusion (*n =* 3 mice per group). Scale bar: 200 μm. (**c**) Representative images of TUNEL staining (red) in intestinal tissues (*n =* 3 mice per group). Scale bar: 300 μm. (**d**) Quantification of TUNEL-positive cells (NC, IR, SSO, T, T + I, O) (*n =* 3 mice per group). (**e**) Quantification of TUNEL-positive cells (NC, IR, SSO, SP) (*n =* 3 mice per group). Groups: NC, sham-operated control; IR, ischemia–reperfusion; SSO, IR + CD36 inhibitor; SP, IR + SP600125 (JNK inhibitor); T, *Thbs1*^flox/flox^/*Ly6g*-CreERT2^+^ control; T + I, *Thbs1*^flox/flox^/*Ly6g*-CreERT2^+^ + IR; O, *Thbs1*^flox/flox^/*Ly6g*-CreERT2^+^ + IR + oxLDL (CD36 agonist). ^*^P < 0.05, ^**^P < 0.01, and ^***^P < 0.001

Collectively, these results reveal that IR-induced JNK activation occurs specifically in IECs and precedes apoptosis. Furthermore, JNK serves as a critical downstream effector of the THBS1–CD36 axis, and its inhibition effectively protects against IR-induced epithelial cell death.

### Neutrophil-derived Thrombospondin-1 induces intestinal epithelial cell apoptosis via the CD36–JNK pathway

To determine whether neutrophil-derived THBS1 induces IEC apoptosis through the CD36–JNK pathway, we established six experimental groups: control (NC), IR, IR + CD36 inhibitor SSO (S), *Thbs1*^flox/flox^/*Ly6g*-CreERT2^+^ control (T), *Thbs1* knockout + IR (T + I), and *Thbs1*^flox/flox^/*Ly6g*-CreERT2^+^ + IR + CD36 agonist oxLDL (O). All CreERT2-positive mice underwent tamoxifen-induced neutrophil-specific *Thbs1* knockout. After intestinal IR treatment (45 min ischemia/3 h reperfusion), neutrophils were isolated from the peripheral blood of each group and cocultured with MODE-K cells for 24 h *in vitro* (Figure 8a). Functional analysis revealed that coculture with IR-group neutrophils significantly suppressed MODE-K cell viability (as determined by a CCK-8 assay). This suppression was markedly alleviated in the S and T + I groups. In the O group, oxLDL treatment counteracted this protective effect (Figure 8b). Scratch wound assays revealed that IR-group coculture considerably impaired MODE-K cell migration. Both the S and T + I groups exhibited the recovery of cell migration, whereas the O group exhibited an attenuation of this restorative effect (Figure 8c). IF staining and quantitative analysis revealed a significant reduction in occludin fluorescence intensity in MODE-K cells after coculture with the IR group. Occludin expression was notably restored in the S and T + I groups. In the O group, the CD36 agonist oxLDL reversed the protective effect of THBS1 deletion on the epithelial barrier (Figure 8d and e). Western blot analysis further confirmed that IR-group co-culture significantly upregulated the protein expression of CD36, p-JNK and the inflammatory cytokines IL-6 and TNF-α in MODE-K cells but downregulated the expression of occludin. These aberrant expression patterns were effectively reversed in the S and T + I groups. In the O group, oxLDL intervention restored the activation status of the CD36–JNK signaling pathway (Figure 8f–h).

**Figure 8:**
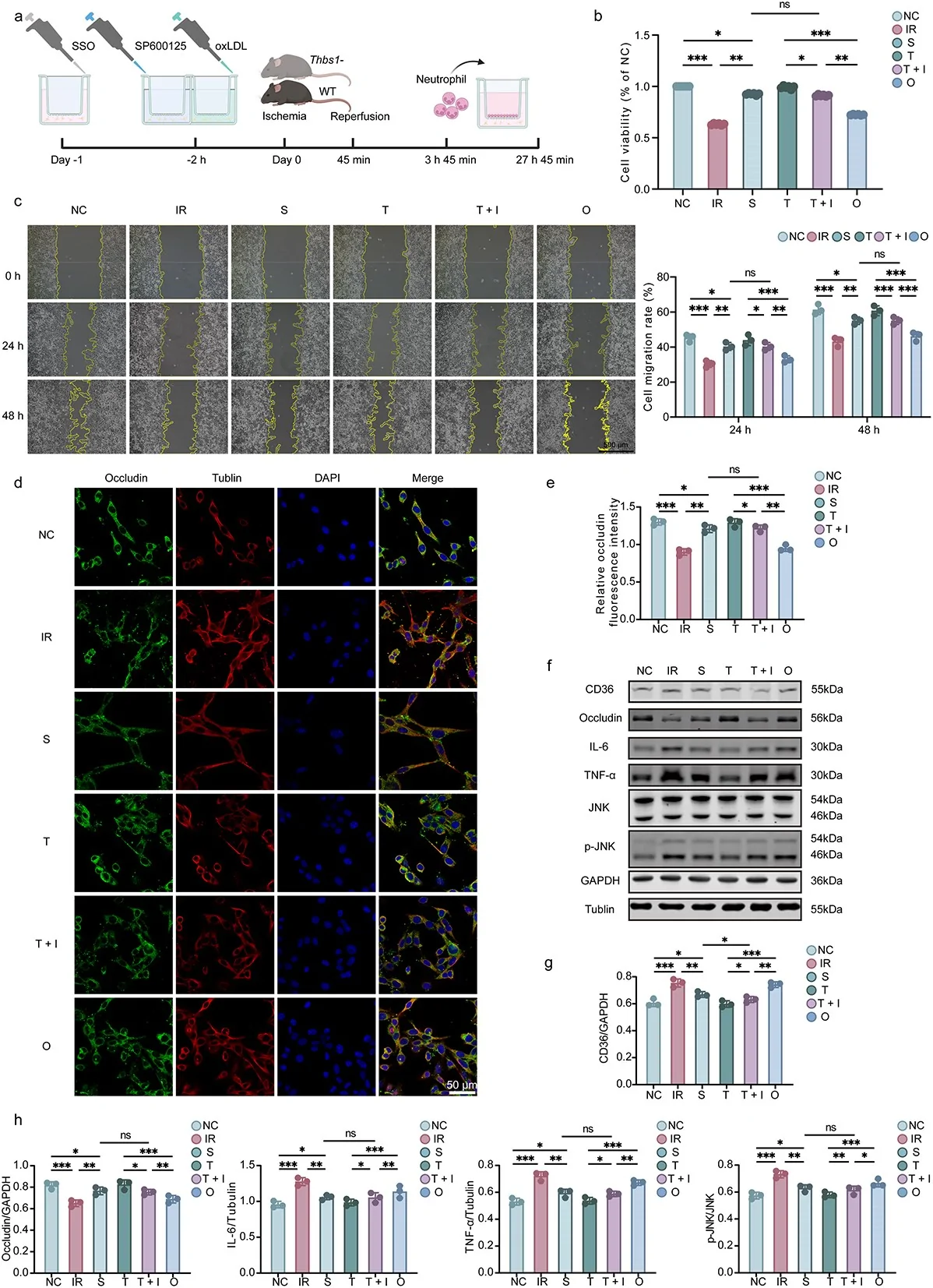
Neutrophil-derived THBS1 induces IEC injury through the CD36–JNK pathway *in vitro*. (**a**) Schematic of the coculture experiment using neutrophils from pharmacologically or genetically modulated mice. (**b**) MODE-K cell viability measured by CCK-8 assay (*n =* 6 biological replicates). (**c**) Wound healing assay (left) and quantification of migration rate (right) (*n =* 3 biological replicates). Scale bar: 500 μm. (**d**) IF staining of occludin (green) in MODE-K cells after coculture, nuclei are stained with DAPI (blue) (*n =* 3 biological replicates). Scale bar: 50 μm. (**e**) Quantification of occludin IF intensity (*n =* 3 biological replicates). (**f**) Western blot analysis of CD36, p-JNK, occludin, IL-6, and TNF-α expression in MODE-K cells (*n =* 3 biological replicates). (**g**) Densitometric quantification of CD36 expression (*n =* 3 biological replicates). (**h**) Densitometric quantification of p-JNK, occludin, IL-6, and TNF-α expression (*n =* 3 biological replicates). Groups: NC, cocultured with neutrophils from sham-operated mice (no drug); IR, cocultured with neutrophils from IR (ischemia–reperfusion)-injured mice (no drug); S, cocultured with neutrophils from IR-injured mice, with SSO (CD36 inhibitor) added to the culture medium; T, cocultured with neutrophils from *Thbs1*^flox/flox^/*Ly6g*-CreERT2^+^ control mice (no drug); T + I, cocultured with neutrophils from *Thbs1*^flox/flox^/*Ly6g*-CreERT2^+^ + IR mice (no drug); O, cocultured with neutrophils from *Thbs1*^flox/flox^/*Ly6g*-CreERT2^+^ + IR mice, with oxLDL (CD36 agonist) added to the culture medium. ^*^P < 0.05, ^**^P < 0.01, and ^***^P < 0.001

The results of apoptosis assays further validated the biological function of this pathway. Annexin V/PI flow cytometry revealed that coculture with neutrophils from the IR group significantly increased the apoptosis rate of MODE-K cells, whereas both SSO treatment and *Thbs1* knockout markedly reduced the proportion of apoptotic cells (Figure 9a and b). To evaluate the role of JNK in apoptosis initiation, we included a JNK inhibitor group that was based on our original experimental groups. Statistical analysis revealed that JNK inhibitor treatment also significantly reduced IR-induced apoptosis, with a protective effect similar to that of SSO treatment (Figure 9b). Notably, oxLDL treatment completely reversed the antiapoptotic effect conferred by *Thbs1* knockout (Figure 9b). TUNEL staining further corroborated these observations. IF staining revealed that co-culture with IR group neutrophils induced pronounced apoptosis, whereas SSO treatment and *Thbs1* knockout markedly decreased the number of TUNEL-positive cells (Figure 9c and d). JNK inhibitor treatment also significantly reduced apoptosis levels, with a protective effect similar to that of SSO treatment (Figure 9d). Consistent with the flow cytometry results, oxLDL treatment partially restored apoptosis in the *Thbs1* knockout background (Figure 9d).

**Figure 9:**
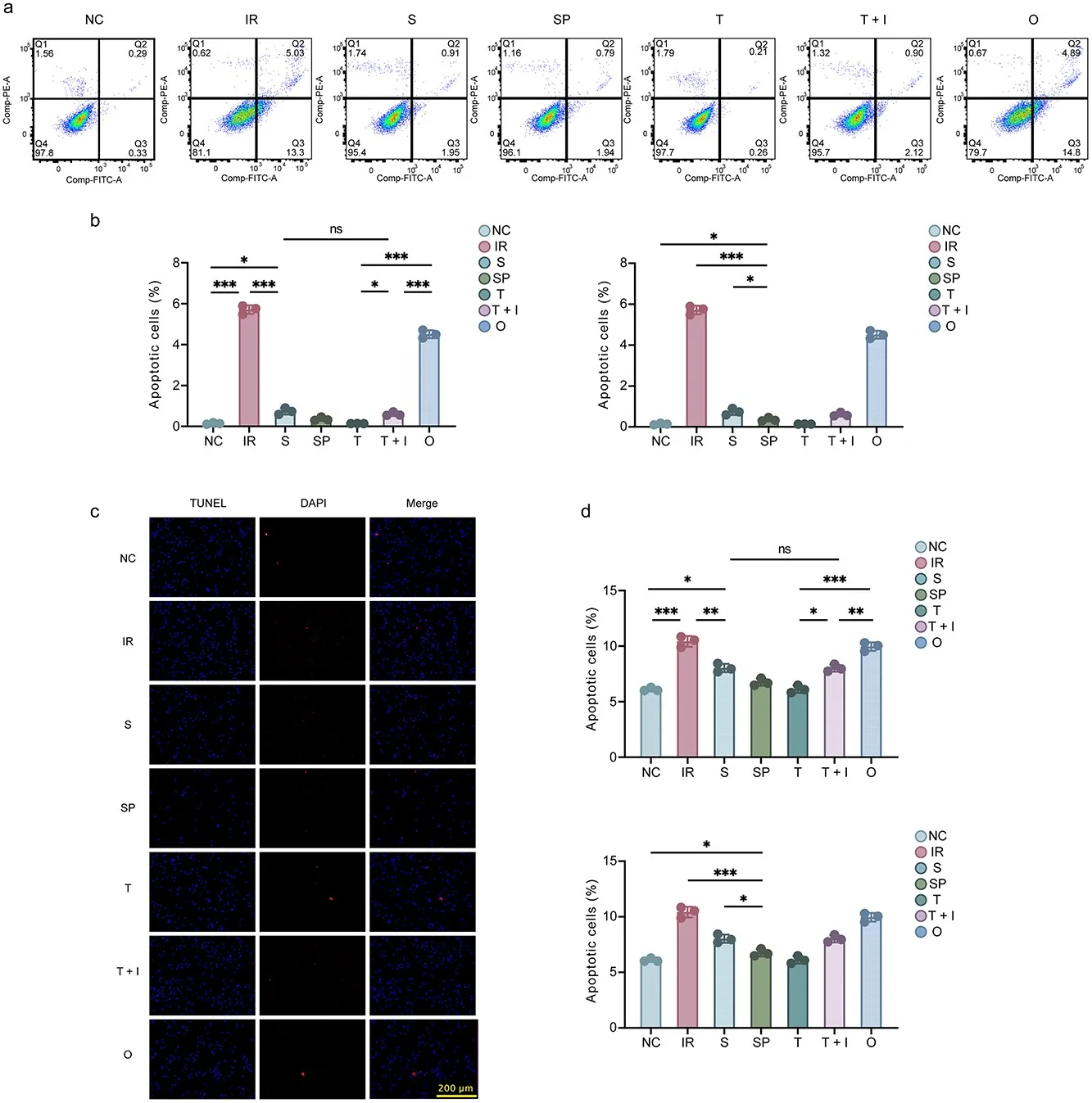
The THBS1–CD36–JNK axis mediates neutrophil-induced MODE-K apoptosis. (**a**) Annexin V/PI flow cytometry plots of apoptotic MODE-K cells after coculture (*n =* 3 biological replicates). (**b**) Quantification of the apoptosis rate from flow cytometry (left: NC, IR, SSO, T, T + I, O; right: NC, IR, SSO, SP) (*n =* 3 biological replicates). (**c**) TUNEL staining (red) of MODE-K cells after coculture. Nuclei are stained with DAPI (blue) (*n =* 3 biological replicates). Scale bar: 200 μm. (**d**) Quantification of TUNEL-positive cells (left: NC, IR, SSO, T, T + I, O; right: NC, IR, SSO, SP) (*n =* 3 biological replicates). Groups: NC, cocultured with neutrophils from sham-operated mice (no drug); IR, cocultured with neutrophils from IR (ischemia–reperfusion)-injured mice (no drug); S, cocultured with neutrophils from IR-injured mice, with SSO (CD36 inhibitor) added to the culture medium; T, cocultured with neutrophils from *Thbs1*^flox/flox^/*Ly6g*-CreERT2^+^ control mice (no drug); T + I, cocultured with neutrophils from *Thbs1*^flox/flox^/*Ly6g*-CreERT2^+^ + IR mice (no drug); O, cocultured with neutrophils from *Thbs1*^flox/flox^/*Ly6g*-CreERT2^+^ + IR mice, with oxLDL (CD36 agonist) added to the culture medium. ^*^P < 0.05, ^**^P < 0.01, and ^***^P < 0.001

Collectively, these findings indicate that neutrophil-derived THBS1 activates the CD36–JNK signaling axis in epithelial cells, progressively impairing cell viability and migration, disrupting barrier integrity, and ultimately inducing apoptosis. As a key mediator in this pathway, CD36 represents a potential therapeutic target, and JNK serves as the core downstream executioner. Targeting this pathway may offer a promising strategy for mitigating IR-induced intestinal mucosal injury.

## Discussion

Damage to the intestinal mucosal barrier can lead to bacterial translocation and systemic inflammatory responses and serves as a critical contributor to multiple organ dysfunction [21, 42]. However, the specific intercellular communication mechanisms regulating IEC injury remain incompletely understood. The mechanisms underlying intestinal IR injury are complex and involve multiple types of intercellular communication. In this study, by integrating unbiased single-cell transcriptomic analysis with functional validation, we revealed a previously unrecognized neutrophil–epithelial cell cross-talk pathway. Initial scRNA-seq analysis revealed a significant expansion of intestinal neutrophils following IR, a finding corroborated in human IR samples, and more importantly, through systematic cell–cell communication analysis, we identified the *Thbs1*–*Cd36* ligand–receptor pair as a specific interaction between neutrophils and IECs. To investigate the downstream signaling of this axis, we integrated multiomics data: transcriptomic analysis of intestinal tissues from *Cd36* global knockout mice revealed that genes whose expression was downregulated were enriched in inflammatory response-related pathways, whereas genes whose expression was upregulated in IR-injured epithelial cells were enriched in the JNK and apoptosis signaling pathways. Further cross-analysis revealed *Mapk8* (JNK1) as a core hub gene. These systematic multiomics data collectively revealed that neutrophil-derived THBS1 activation of JNK proapoptotic signaling via epithelial CD36 is a promising mechanism that warrants functional validation.

IR injury is a common pathological process leading to intestinal mucosal barrier disruption in clinical practice and frequently occurs in critical conditions such as shock and burns. In this study, we used a mouse model of 45 min of ischemia followed by 3 h of reperfusion to focus on the early acute phase of IR injury. The selection of these time points was based on previous studies: 45 min of ischemia induces stable and severe mucosal injury while ensuring relatively low mortality [43, 44], and 3 h of reperfusion represents an optimal time point for capturing early apoptotic events, when key apoptotic markers become detectable but before later-phase repair and secondary inflammation confound the assessment of acute injury mechanisms [45–47].

Neutrophils are among the first immune cells to infiltrate the intestinal mucosa following IR injury and play a critical role in barrier disruption; however, the specific mechanisms through which they direct the fate of epithelial cells remain poorly understood. As central effectors in IR injury, neutrophils exert their pathogenic effects through diverse and complex mechanisms. Previous studies have demonstrated that neutrophils disrupt the intestinal epithelial barrier primarily through four pathways. The first pathway is ROS-mediated oxidative damage: superoxide anions and hydrogen peroxide generated during respiratory bursts directly attack membrane lipids and proteins, inducing oxidative stress, mitochondrial dysfunction, and apoptosis in epithelial cells [48]. The second pathway is inflammatory cytokine-induced death signals: TNF-α activates the caspase cascade via TNFR1 to directly induce apoptosis while also activating the NF-κB pathway to amplify inflammatory responses and promote further neutrophil infiltration [49]; IL-1β and other cytokines further exacerbate the inflammatory cascade [50]. The third pathway is protease-mediated matrix degradation: neutrophils release elastase that directly cleaves extracellular matrix components and epithelial junction proteins, disrupting basement membrane integrity and epithelial polarity, which leads to cell detachment [51]. The fourth pathway is NET-mediated cytotoxicity: studies have shown that NETs induce epithelial cell necroptosis through the TLR4/RIPK3/FUNDC1 pathway [52]; furthermore, histones within NETs exhibit direct cytotoxicity, with NETs serving as physical scaffolds that capture and activate additional immune cells [53]. To confirm the core pathogenic role of neutrophils in our model, we depleted neutrophils *in vivo* using an anti-Ly6G antibody. The results showed that neutrophil depletion significantly ameliorated IR-induced intestinal shortening, tissue edema, mucosal structural damage, and loss of the barrier protein occludin. These findings demonstrate that neutrophils are key drivers of IR-induced intestinal structural disruption, barrier dysfunction, and tissue edema. Therefore, identifying the specific molecular mechanisms through which these cells directly induce the death of epithelial cells becomes a critical question.

To investigate the mechanisms through which neutrophils induce intestinal mucosal injury, our study focused on the THBS1–CD36–JNK axis, as predicted by bioinformatics analysis. Previous studies have demonstrated that the inhibitory effect of THBS1 on hepatocyte lipid synthesis is completely abolished upon *Cd36* knockout [54]; furthermore, neonatal intestinal defense studies have confirmed the functional coupling of THBS1 and CD36 co-expression in the intestinal epithelium during pathogen clearance [55]. These independent investigations across different organs and physiopathological contexts collectively indicate that the THBS1–CD36 axis represents a fundamental and conserved core mechanism mediating intercellular communication. To clarify the cellular source and necessity of THBS1, we generated mice with a neutrophil-specific *Thbs1* knockout. Compared with WT IR mice, knockout mice exhibited significantly ameliorated intestinal injury, barrier dysfunction, and inflammatory responses, demonstrating that neutrophil-derived THBS1 is an indispensable factor driving epithelial injury. Further investigation revealed that *Thbs1* knockout resulted in significantly decreased CD36 expression in the intestinal epithelium, suggesting that THBS1 may positively regulate CD36 expression. *In vitro* coculture experiments similarly confirmed that the cytotoxic effects of IR-derived neutrophils on epithelial cells depend on their expression of THBS1. These results preliminarily establish an association between THBS1 and CD36; however, whether CD36 functions as a downstream receptor of THBS1 requires further pharmacological validation. In this study, single-cell sequencing and cell–cell communication analysis revealed that the THBS1–CD36 ligand–receptor pair exhibited IR specificity—significant interactions with neutrophils as the ligand source and IECs as the receptor were detected exclusively in the IR injury group, while this pathway remained quiescent under physiological conditions. These findings reveal the ligand-dependent nature of this axis: Although IECs constitutively express high levels of CD36 for fatty acid absorption, the death pathway remains inactive because of the absence of the THBS1 ligand from immune cells. To directly validate the pathogenic potential of THBS1, we stimulated IECs with purified THBS1 protein. The results revealed that THBS1 treatment alone for 2 h significantly downregulated barrier protein expression and upregulated inflammatory cytokine expression. Notably, IR injury not only provides the ligand—THBS1 released by infiltrating neutrophils—but also induces further significant upregulation of CD36 expression in IECs, as we observed. The upregulated expression of this receptor may increase epithelial cell sensitivity to THBS1, together constituting dual activation conditions of ligand emergence and receptor upregulation. Therefore, THBS1–CD36 axis activation exhibits a strict environmental dependence, requiring the specific pathological microenvironment created by IR—neutrophil infiltration providing the ligand and upregulated expression of epithelial CD36 increasing the sensitivity—as activation conditions rather than representing the constitutive signaling of CD36 itself.

Building upon the established THBS1–CD36 association, we further defined the functional role of CD36 downstream of THBS1 using pharmacological approaches. CD36 inhibition phenocopied the protective effects of THBS1 deficiency, whereas CD36 agonist treatment reversed the protection conferred by *Thbs1* knockout. This bidirectional validation not only demonstrates the necessity of CD36 but also confirms that its activation is sufficient to mediate the pathogenic effects of THBS1, providing rigorous causal evidence for the THBS1–CD36 axis. The terminal event of this signaling axis was localized to JNK activation. To elucidate the temporal relationship and cellular source of JNK activation in relation to epithelial apoptosis, we performed IF colocalization analysis at various time points following IR injury. The results revealed that p-JNK-positive signals were detectable as early as 5 min after reperfusion, whereas TUNEL-positive cells appeared at 30 min after reperfusion, confirming that JNK activation precedes epithelial apoptosis. Furthermore, colocalization staining of p-JNK, TUNEL and the epithelial marker E-cadherin revealed that p-JNK and TUNEL signals were primarily localized within E-cadherin-positive IECs, indicating that IR-induced JNK activation occurs specifically in the intestinal epithelium. On the basis of these findings, we observed that JNK phosphorylation levels were coordinately regulated by CD36 and THBS1: blocking CD36 or knocking out *Thbs1* suppressed JNK activation, whereas treatment with a CD36 agonist restored JNK phosphorylation while reversing the protective phenotype. More importantly, direct JNK inhibition effectively attenuated IR-induced apoptosis, further suggesting JNK as the key kinase that initiates apoptotic signaling downstream of the THBS1–CD36 axis. By integrating these genetic and pharmacological findings, we systematically elucidated a complete signaling cascade from neutrophil-derived THBS1 through epithelial CD36 to JNK activation. On the basis of the literature, we speculate that the THBS1–CD36–JNK axis may undergo a functional transition during the repair phase. First, JNK signaling itself exhibits duality [56], with excessive JNK activation promoting apoptosis, and moderate JNK activity participating in cell migration and wound healing. In IECs, JNK facilitates cell migration by regulating actin dynamics and focal adhesion turnover [57]. Therefore, residual JNK activation in the late phase of IR may shift to participate in epithelial re-epithelialization. Second, THBS1 can mediate opposing functions through different receptors. While our study demonstrated that THBS1 promotes apoptosis via CD36-mediated JNK activation, THBS1 can also exert prorepair effects through CD47 and TGF-β receptors. Studies have shown that THBS1–CD47 signaling promotes endothelial and epithelial cell migration [33] and that THBS1-mediated activation of latent TGF-β facilitates matrix deposition and tissue remodeling [58]. Thus, during the late phase of IR, as neutrophil infiltration subsides and macrophages become the predominant cell type, the source of THBS1 may shift from neutrophils to macrophages or fibroblasts, and its effector receptor may similarly shift from CD36 to CD47 or TGF-β, thereby transitioning from proapoptotic to prorepair functions. Furthermore, THBS1 may participate in IR late-phase repair through cross-activation of other repair-related pathways. The THBS1 protein contains a TSR domain homologous to that found in other Wnt signaling agonists, such as the R-spondin family [59], suggesting its potential to regulate Wnt signaling, although this speculation awaits experimental validation. Previous studies have confirmed that THBS1 can activate the ERK pathway through multiple mechanisms. For instance, in vascular endothelial cells, THBS1 indirectly promotes ERK phosphorylation through HIF-1/VEGF signaling [60]; in fibroblasts, THBS1 interacts with cell surface calreticulin to activate ERK via Gi protein- and PI3K-dependent pathways, thereby regulating cytoskeletal reorganization and adhesion [61]. These findings suggest that they may serve as important downstream mechanisms through which THBS1 promotes tissue repair during the late phase of IR.

In addition to validating the injury caused by neutrophil–epithelial cell interactions, we also performed bioinformatics predictions regarding the upstream promoter region of *Thbs1* in neutrophils. Our bioinformatic analysis revealed a notable candidate transcription factor, v-maf musculoaponeurotic fibrosarcoma oncogene homolog K (MAFK), a member of the small Maf family, that is involved in oxidative stress responses and apoptosis regulation. Studies have shown that MAFK overexpression accelerates IEC apoptosis by promoting caspase-3 cleavage, thereby exacerbating intestinal inflammatory injury [62]. Recent research has also implicated MAFK in the regulation of pyroptosis [63]. Given that oxidative stress and cell death are central pathological features of IR injury, the possibility of MAFK serving as an upstream regulator of *Thbs1* warrants attention. On the basis of the above analysis, we propose a more comprehensive pathological cascade model for IR injury. In the early stage of IR injury, hypoxia and the release of initial DAMPs may activate transcription factors such as MAFK, promoting the transcription and release of *Thbs1* from neutrophils. THBS1 then induces epithelial cell apoptosis via the CD36–JNK axis, and DAMPs released from early apoptotic cells further activate more neutrophils, amplifying the inflammatory storm of ROS, inflammatory cytokines, and NETs. This three-level cascade model—transcription factor initiation, ligand–receptor execution, and inflammatory storm amplification—provides a new perspective for understanding the complete pathological process of IR injury. Future studies using ChIP–qPCR and luciferase reporter assays are warranted to validate the direct transcriptional regulation of *Thbs1* by MAFK.

In summary, through multiomics screening and multilevel functional experiments in this study, we systematically elucidated a complete signaling axis comprising neutrophil THBS1, epithelial CD36, and JNK in intestinal IR injury. This pathway reveals that activated neutrophils can directly activate the JNK apoptotic program in epithelial cells by releasing THBS1, establishing that neutrophil-derived soluble ligands directly regulate epithelial cell apoptosis as a pivotal event in IR injury. These findings, together with existing damage theories concerning ROS, inflammatory cytokines, proteases and NETs, constitute a more comprehensive pathophysiological landscape. Furthermore, these findings suggest that this specific ligand–receptor pair-mediated regulation of remote cell death may have broader implications for the maintenance of physiological homeostasis and the occurrence of pathological injuries.

This study had several limitations. First, the spatial resolution of our single-cell RNA-seq and CellChat analyses was limited. Although we verified the interaction between THBS1 and CD36 through IF colocalization and Co-IP, higher-resolution spatial omics profiling remains to be performed in future studies. Second, we focused primarily on the early stage of IR injury, and the potential role of the THBS1–CD36 axis in the late repair phase remains to be explored. Third, although we confirmed the direct pathogenic potential of THBS1 through *in vitro* stimulation with purified THBS1 protein, whether exogenous THBS1 administered via different routes activates this pathway requires further investigation. Despite these limitations, our findings provide new therapeutic insights for critical conditions such as shock and burns, in which prognosis is largely determined by intestinal barrier integrity. Targeting this axis with neutralizing antibodies or inhibitors holds promise as a potential translational strategy for protecting the intestinal mucosa and preventing disease progression.

## Conclusions

In this study, we systematically elucidated a complete pathogenic pathway in intestinal IR injury, spanning from intercellular recognition to the initiation of damage. By integrating unbiased single-cell transcriptomic screening with mechanistic validation, we confirmed that infiltrating neutrophils release THBS1 as a key mediator, thereby activating the proapoptotic CD36–JNK signaling axis in IECs. Using a neutrophil-specific gene knockout model, we demonstrated that neutrophil-derived THBS1 is a necessary driver of epithelial apoptosis, as its absence significantly alleviated IR-induced epithelial damage. Through mechanistic dissection, we identified CD36 as the critical signaling receptor for THBS1 in epithelial cells, with JNK activation serving as the terminal executor of this apoptotic program. Moreover, pharmacological inhibition of CD36 protects barrier integrity while its specific activation reverses the protective effect of *Thbs1* knockout, we established the translational potential of targeting this signaling axis. Collectively, these findings establish the THBS1–CD36–JNK pathway as a previously underappreciated core mechanism in IR injury. This insight is particularly relevant for severe conditions such as burns, shock, and intestinal transplantation, in which clinical outcomes are determined by epithelial barrier function. Future research aimed at selectively inhibiting this neutrophil–epithelial signaling axis while preserving the physiological repair functions of Thbs1 in other contexts may yield novel therapeutic strategies for protecting intestinal barrier function in critical illnesses.

## Supplementary Material

Supplementary_material_tkag059
