# Supplementary material for: A neutrophil–epithelial thrombospondin‑1–CD36–c‑Jun N‑terminal kinase axis orchestrates mucosal barrier failure in intestinal ischemia–reperfusion
Source: Burns Trauma. 2026 Sep 2;14:tkag059. doi: 10.1093/burnst/tkag059 (PMC13654182; doi:10.1093/burnst/tkag059)
Supplement: Supplementary file 1 [file supplementary_material_tkag059.zip › Caption_suppl.docx]

**Supplementary Figure Legends**

**Figure S1.**

The schematic diagram of neutrophils causing intestinal mucosal injury through the Thbs1-CD36-JNK axis. (a) Shell plot of communication strength among different cell subpopulations. (b) Circle plot of THBS signaling strength in NC and IR groups. (c) Bubble plot of ligand–receptor pairs with neutrophils as ligands and other cells as receptors. (d) Volcano plot of differential analysis from intestinal bulk RNA-seq of CD36 KO mice.

**Figure S2.**

Co-IP analysis of THBS1-CD36 interaction. MODE-K cells were cocultured with neutrophils from IR-injured mice. Following coculture and extensive washing, cell lysates were immunoprecipitated with anti-CD36 antibody or normal rabbit IgG (negative control). Immunoprecipitates and input samples were immunoblotted with anti-THBS1 and anti-CD36 antibodies. Representative blots from three independent experiments are shown (n=3 biological replicates).

**Figure S3.**

Validation of anti-Ly6G antibody treatment on intestinal homeostasis. (a) Representative images of intestinal length (n=6 mice per group). (b) Quantification of intestinal length (n=6 mice per group). (c) Intestinal wet/dry weight ratio (n=6 mice per group). (d) Western blot analysis of Occludin, IL-6, and TNF-α expression (n=3 mice per group). (e) Densitometric quantification of Western blot results (n=3 mice per group). Groups: NC, untreated control; 1A8, mice treated with anti-Ly6G monoclonal antibody (clone 1A8) for neutrophil depletion; 2A3, mice treated with isotype control antibody (clone 2A3)

**Figure S4.**

Flow cytometry analysis confirming neutrophil depletion efficiency following anti-Ly6G treatment.

**Figure S5.**

Direct effects of Thbs1 protein on intestinal epithelial cells. (a) MODE-K cell viability measured by CCK-8 assay (n=6 biological replicates). (b) Western blot analysis of Occludin, IL-6 and TNF-α expression (n=3 biological replicates). (c) Densitometric quantification of Occludin expression (n=3 biological replicates). (d) Densitometric quantification of IL-6 expression (n=3 biological replicates). (e) Densitometric quantification of TNF-α expression (n=3 biological replicates). Groups: NC, cocultured with neutrophils from sham-operated mice; IR, cocultured with neutrophils from IR (ischemia-reperfusion)-injured mice; TP, cocultured with neutrophils from IR-injured mice with recombinant Thbs1 protein added to the culture medium; T, cocultured with neutrophils from Thbs1^flox/flox^/Ly6G-CreERT2^+^ control mice; T+I, cocultured with neutrophils from Thbs1^flox/flox^/Ly6G-CreERT2^+^ + IR mice.

**Figure S6.**

Genotype identification and Thbs1 knockout validation in neutrophil-specific Thbs1 conditional knockout mice. (a) Gel electrophoresis of PCR genotyping for Thbs1-floxed and Ly6g-2A-CreERT2 alleles. (b) Western blot analysis of Thbs1 protein expression in control and cKO mice.

**Figure S7.**

Flow cytometry validation of neutrophil purity in mouse peripheral blood. (n=3 mice per group)

**Figure S8.**

Flow cytometry gating strategy for IEC isolation. Representative plots showing the sequential gating strategy to identify EpCAM⁺CD45⁻ intestinal epithelial cells. Data are representative of three independent experiments.

**Figure S9.**

Flow cytometry gating strategy for intestinal epithelial cell (IEC) isolation. Groups: NC, cocultured with neutrophils from sham-operated mice (no drug); IR, cocultured with neutrophils from IR (ischemia-reperfusion)-injured mice (no drug); S, cocultured with neutrophils from IR-injured mice, with SSO (CD36 inhibitor) added to the culture medium; T, cocultured with neutrophils from Thbs1^flox/flox^/Ly6G-CreERT2^+^ control mice (no drug); T+I, cocultured with neutrophils from Thbs1^flox/flox^/Ly6G-CreERT2^+^ + IR mice (no drug); O, cocultured with neutrophils from Thbs1^flox/flox^/Ly6G-CreERT2^+^ + IR mice, with oxLDL (CD36 agonist) added to the culture medium.
